# Design, synthesis, crystallization and biological evaluation of new symmetrical biscationic compounds as selective inhibitors of human Choline Kinase α1 (ChoKα1)

**DOI:** 10.1038/srep23793

**Published:** 2016-03-31

**Authors:** Santiago Schiaffino-Ortega, Eleonora Baglioni, Elena Mariotto, Roberta Bortolozzi, Lucía Serrán-Aguilera, Pablo Ríos-Marco, M. Paz Carrasco-Jimenez, Miguel A. Gallo, Ramon Hurtado-Guerrero, Carmen Marco, Giuseppe Basso, Giampietro Viola, Antonio Entrena, Luisa Carlota López-Cara

**Affiliations:** 1 Departamento de Química Farmacéutica y Orgánica, Facultad de Farmacia, Campus de Cartuja, 18071 Granada (Spain); 2 Departamento de Bioquímica y Biología Molecular I, Facultad de Ciencias, Campus Fuentenueva, 18071 Granada (Spain); 3 Dipartimento di Salute della Donna e del Bambino, Laboratorio di Oncoematologia Pediatrica, Università di Padova, Padua, Italy; 4 Institute of Biocomputation and Physics of Complex Systems (BIFI), University of Zaragoza, BIFI-IQFR (CSIC) Joint Unit, Mariano Esquillor s/n, Campus Rio Ebro, Edificio I+D; Fundacion ARAID, Edificio Pignatelli 36 (Spain)

## Abstract

A novel family of compounds derivative of 1,1′-(((ethane-1,2-diylbis(oxy))bis(4,1-phenylene))bis(methylene))-bispyridinium or –bisquinolinium bromide (**10a-l**) containing a pair of oxygen atoms in the spacer of the linker between the biscationic moieties, were synthesized and evaluated as inhibitors of choline kinase against a panel of cancer-cell lines. The most promising compounds in this series were 1,1′-(((ethane-1,2-diylbis(oxy))bis(4,1-phenylene))bis(methylene))bis(4-(dimethylamino)pyridinium) bromide (**10a**) and 1,1′-(((ethane-1,2-diylbis(oxy))bis(4,1-phenylene))bis(methylene))-bis(7-chloro-4-(pyrrolidin-1-yl)quinolinium) bromide (**10l**), which inhibit human choline kinase (ChoKα1) with IC_50_ of 1.0 and 0.92 μM, respectively, in a range similar to that of the previously reported biscationic compounds **MN58b** and **RSM932A**. Our compounds show greater antiproliferative activities than do the reference compounds, with unprecedented values of GI_50_ in the nanomolar range for several of the cancer-cell lines assayed, and more importantly they present low toxicity in non-tumoral cell lines, suggesting a cancer-cell-selective antiproliferative activity. Docking studies predict that the compounds interact with the choline-binding site in agreement with the binding mode of most previously reported biscationic compounds. Moreover, the crystal structure of ChoKα1 with compound **10a** reveals that this compound binds to the choline-binding site and mimics **HC-3** binding mode as never before.

Cancer is a worldwide health threat and the second leading cause of mortality in developed countries^1^,^2^. Since many of the current treatments still prove toxic and/or lead to drug resistance, there is a strong demand for the discovery and development of effective new cancer therapies^3^.

Protein kinases have emerged as one of the most important types of targets in cancer-drug discovery due to their major roles in regulating cell growth and survival and many other cell functions^4^,^5^. An abnormal kinase signaling network underlies the development and progression of tumors, and thus the targeted inhibition of protein kinases has become an attractive strategy in cancer treatment (for a recent review see Gross *et al.*^6^) and in the last decade the intense development in the field has led to different kinase inhibitors that have been approved for use in clinical therapy.

Choline kinase (ChoK) (EC 2.7.1.32) catalyzes the phosphorylation of choline by ATP in the presence of Mg^2+^ to yield phosphocholine (PCho) and ADP^7^,^8^. This step introduces choline to the so-called Kennedy or CDP-choline pathway for the biosynthesis of phosphatidylcholine, which represents the most abundant class of phospholipids in eukaryotic cells, constituting 40–60% of the phospholipids content in cell membranes^9^. In addition to forming the major structural component of the membrane bilayer, phosphatidylcholine also serves as a precursor for the production of lipid second messengers^10^.

Mammalian ChoK exists as three isoforms, encoded by two separate genes^11^,^12^. In humans, ChoKα1 (457 amino acids) and ChoKα2 (439 amino acids) derive from a single gene (*chk-α*) by alternative splicing, while ChoKβ (395 amino acids) is the product of a different gene (*chk-β*). The amino acid sequence identity is 56% between ChoKα and ChoKβ, and both *chk-α* and *chk-β* mRNAs, as well as their encoded protein isoforms, are ubiquitously expressed in diverse tissues^13^. Each isoform is present as either dimers (homo- or hetero-) or as tetramers in solution and is not active in monomeric form^7^, suggesting that, for higher eukaryotes, dimeric ChoK is the minimum functional form.

Choline kinase is overexpressed in many tumors such as breast, lung, bladder, colon, prostate, ovary, and liver carcinomas^14^,^15^,^16^ and recently elevated enzymatic activity has also been shown in T-lymphoma^17^. This increasing expression leads to abnormal choline metabolism, resulting in higher phosphocholine levels, which refer to a cholinic phenotype associated with oncogenesis and tumor progression^18^. As a result, ChoKα, has become an attractive target for novel anticancer therapies.

The determination of the crystal structures of ChoK proteins from *Caenorhabditis elegans* and *human*, in which two monomers were dimerized in each asymmetric unit^19^,^20^ and the correct identification of ATP and choline binding sites into crystal structure of human ChoKα2 isoform, have enabled the design and the synthesis of a series of asymmetrical molecules as potential ChoK inhibitors and antiproliferative compounds^21^,^22^. Figure 1 shows **HC-3**, the first inhibitor of choline kinase described, **MN58b** and RSM923A, which belong to the first generation of Chok inhibitors^23^,^24^,^25^,^26^,^27^,^28^, and the most promising compounds developed by our group. Note that **RSM932A** (also called TCD-717) has evidenced a low-toxicity profile with improved tolerability in mice^29^ and a Phase I clinical trial has just been completed for the treatment of advanced solid tumors (http://clinicaltrials.gov/ct2/show/NCT01215864).

For compounds **1** and **2**, we identified the adenine and 1-benzyl-4-(dimethylamino)pyridinium as the most efficient fragments of these molecules by the deconvolution approach based on the ChoKα1/1 (PDB ID: 3ZM9)^30^ and ChoKα1/**2** (PDB ID: 4BR3)^31^ crystal structures, demonstrating that the adenine fragment occupies the ATP binding site and that the pyridinium fragment, through its positive charge delocalized over the nitrogen atom, mimics the positive charge present in choline or in **HC-3**.

The second generation of inhibitors (compounds **3** and **4**), asymmetrical bispyridinium compounds, proved to be good inhibitors and provide the discovery of a new inhibitory binding site on ChoKα1 Compound **4** (Fig. 1), which induced the opening of new adjacent binding site where the 4-Chloro*-N*-methylaniline fragment is located, adopting an unprecedented modality of binding to ChoKα1(ChoKα1/**4**, PDB ID: 4CG8)^32^, while compound **3** (with biphenyl group as a linker) adopts a binding mode similar to the one observed for compound **2**.

In an effort to produce additional highly active compounds, we focused on longer spacers between biphenilic or bipyridinic rings, which have electron donor or acceptor groups necessary to increase the binding to the enzyme through of hydrogen bonds and the solubility, while retaining some inhibition properties.

Deep modeling and virtual screening studies^32^,^33^,^34^ have suggested the interaction with the choline binding site in the ChoKα1/**4** complex while keeping the biscationic structure unchanged. Thus a classical bioisosteric exchange between carbon and oxygen atoms could increase, on one hand, the polarity and the solubility of these compounds and, on the other hand, the affinity for the enzyme due to the synclinal conformation of the linker of these molecules. In this way, in the present study, we reconfigured the substitution pattern around linker moiety by the preparation of 1,1′-(((ethane-1,2-diylbis(oxy))bis(4,1-phenylene))bis(methylene))-bis[4-pyridinium or quinolinium] bromide derivatives with general structures **10a-l** (Fig. 1). **MN58b**, **RSM932A**, **1**, **2**, **3**, **4**, our recently published compound **5**^35^ (Fig. 1) and the most active compounds described by S. Trousil^36^, were taken as patterns to improve the polarity and solubility, while also improving inhibition by the enzyme and consequently enhancing the antiproliferative effect. This series was obtained by interchanging the substitution pattern of linker by the introduction of two oxygen atoms in the linker, in order to determine the influence of these groups on the antiproliferative and inhibitory activity of ChoKα1, using various cationic heads previously synthesized by our group in similar compounds. We fixed the most successful cationic heads described previously (pyridinium and quinolinium salts)^32^,^35^,^36^,^37^,^38^,^39^,^40^ and examined several 4-substitutions with alkylamines or phenylamines on the arylmoiety. Also, we introduced a quinuclidinium salt, which mimics the trimethylammonium of the choline, but potentially prevents interactions with the cholinergic system^41^.

## Results and Discussion

### Chemistry

Microwave-assisted reactions present several advantages, such as a remarkable reduction in reaction times compared to those of the conventionally heated reactions and often lead to improved yields^42^,^43^. In the present work, we describe the use of microwave irradiation as an energy source for the synthesis of the intermediate (**7** and **8**) of twelve 1,2-bis(*p*-methylphenoxy)ethane derivatives **10a-l**, substituted in the methylphenoxy group with different cationic heads as moieties. These compounds can be also considered as more polar analogues of choline kinase inhibitor derivatives than those previously synthesized.

**Figure 2:**
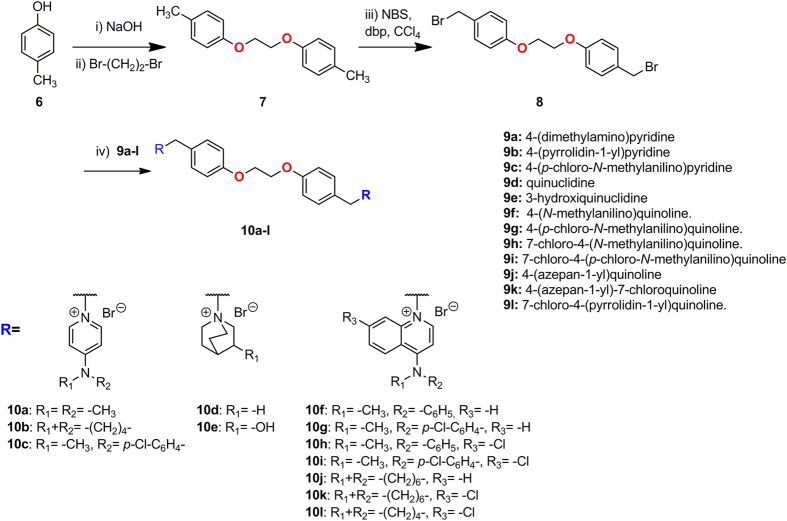
General synthetic pathway followed in the preparation of compounds **10a-l** i) NaOH, EtOH, RT 30 min. ii) Br-(CH_2_)_2_-Br, MW, 130 °C, 28 min. iii) NBS, CCl_4_, dbp, MW 130 °C, 21 min. iv) **10a-l**, CH_3_CN, 72 hours.

The syntheses of compounds **10a-l** is shown in Fig. 2, and follows three easy steps. The first is the treatment of the 4-methylphenol (6) in ethanol with NaOH (1.1 equiv) stirring at room temperature for 30 min, followed by the addition of 1,2-dibromoethane (0.5 equiv), under microwave irradiation (130 °C, 28 min) to provide the 1,2-bis(*p*-methylphenoy)ethane (**7**)^44^,^45^. Then, bromination in the methylene of 7 with NBS and dibenzoylperoxide in CCl_4_ also under microwave irradiation (120 °C, 21 min), to give the 1,2-bis(4-bromomethylphenoy)ethane (**8**)^45^,^46^. In comparison with conventional (thermal) heating, the microwave heating reduced the reaction time in both reactions (30 min vs. 8 h and 21 min vs. 5 h, respectively), but we also noted some yield improvement (35% vs. 21% and 65% vs. 39%, respectively)^45^,^46^.

We conducted different experiments to achieve these successful results with the MW (Table S1 of Supplementary information). Although in the second step, we were restricted by the quantity to use, since using only 100 mg of derivative **7** gave the best yields, while more quantity of **7** led to diminished yields. This result was due to the volume of the reactor, which allows only 5 mL of the mixture, while more than 100 mg of **7** derivative would need more solvent to dissolve it.

Finally, the last step is the introduction of cationic heads (previously synthesized using the procedure reported^32^,^35^,^36^,^37^,^38^,^39^,^40^ by means of a simple S_*N*_2 reaction in acetonitrile under argon atmosphere for 72 h at reflux of 1,2-bis(4-bromomethylphenoxy)ethane (**8**) and the 4-substituted pyridine derivative (**9a-c**), quinuclidine derivative (**9d-e**) or 4-substituted quinoline or 7-Chloro-4-substituted quinoline (**9f-l**) to afford **10a-l** with moderate or good yields^46^.

### Docking studies

Docking studies were made in order to design the new ChoK inhibitors. The crystal structures of greatest interest for docking studies are those of ChoKα1 isoenzyme in complex with compounds **2** (PDB ID: 4BR3)^31^ and **4** (PDB ID: 4CG8)^32^, since the cationic heads of compounds described in this paper are similar to those of compounds **2** and **4**.

**Figure 3:**
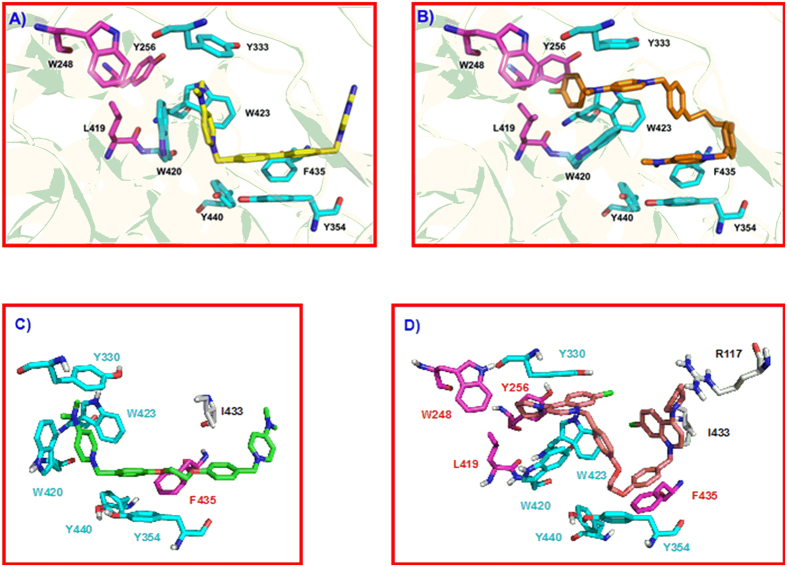
(**A**) Crystal structure of ChoKα1/**2** complex (PDB ID: 4BR3). Compound **2** (carbon atoms in yellow color) is inserted into the Cho binding site (carbon atoms in cyan color). (**B**) Crystal structure of ChoKα1/**4** complex (PDB ID: 4CG8). Compound **4** (carbon atoms in orange color) is inserted into the Cho binding site (carbon atoms in cyan color) and in an additional binding site (carbon atoms in magenta color) that has been open by a conformational change of Tyr256, Tyr333, and Trp420 sidechains, induced by the insertion of compound **4** into the enzyme. (**C**) Resulting pose of compound **10a** (carbon atoms in green color) in the Cho binding site of the ChoKα1/**2** crystal structure, and D) Resulting pose of compound **10l** (**B**, carbon atoms in yellow color) in the ChoK binding site of the ChoKα1/**4** crystal structure.

Figure 3A shows compound **2** (carbon atoms in yellow color) inserted into the Cho binding site, being stabilized by cation-π interactions with Tyr333, Tyr354, Tyr440, Trp420, Trp423, and Phe435 (carbon atoms in cyan color). In particular, the biphenyl group shows optimal parallel hydrophobic stacking interactions with Tyr354, and the 4-(dimethylamino)pyridinium moiety interacts through parallel cation-π interaction with Trp420. The orientation of this compound inside the Cho binding site was accommodated by a conformational change of Tyr333, which moved back to create an extra space^30^,^31^. The adenine fragment of compound 2 inserted into the Cho binding site was outside the enzyme and showed no interaction with the protein (Figure S1 of Supplementary information), 1-(biphenyl-4-ylmethyl)-4-(dimethylamino)pyridinium being the key fragment of this compound for the interaction at the Cho binding site^30^,^31^. Figure 3B shows compound **4** (carbon atoms in orange color) inserted into the ChoKα1 crystal structure. This compound adopts a new different binding mode, inducing a conformational change in some amino acids. Tyr256, Tyr333, and Trp420 are the residues that undergo the major changes, and the rotation of these side chains is critical to allow the insertion of the 4-chloro-*N*-methylaniline fragment into an additional binding site (carbon atoms in magenta color), being stabilized by hydrophobic interactions with Trp248, Tyr256, Tyr333, Leu419, Trp420, and Trp423. The rest of the molecule is located inside the Cho binding site (carbon atoms in cyan color), the pyridinium moieties being stabilized through cation-π interactions with Tyr333, Tyr354, Trp420, and Tyr440^32^. Compound **4** is more inserted into the Cho binding site in comparison to compound **2** (Figure S1 of Supplementary Information) since this compound makes the complete opening of this site.

Docking studies have been performed in both crystal structures and the analysis of the resulting poses indicates which compounds could be similar to compound **2** or to compound **4**. In fact, compounds **10a**, **10b**, **10d**, and **10e** have shown good poses in the crystal structure of compound **2**, while the correct poses of compounds **10c**, **10f**, **10g**, **10h**, **10i**, **10j**, **10k**, and **10l** resulted in the crystal structure of compound **4**.

As an example, Fig. 3C,D show the resulting pose of compounds **10a** and **10l**. Compound **10a** (carbon atoms in green color) has two 4-(dimethylamino)pyridinium cationic heads, similarly to compound **2** (Fig. 3A). One 1-benzyl-4-(dimethylamino)pyridinium fragment is inserted in a way very similar to that of compound **2**: i) the cationic head is situated close to Trp420, being stabilized by π-cation interactions with Trp420, Tyr333, and Trp423; and: ii) the benzyl fragment is also optimized by hydrophobic stacking interactions with Tyr354. The linker of compound **10a** is extremely long, and the second 1-benzyl-4-(dimethylamino)pyridinium fragment is situated outside of the enzyme, an additional hydrophobic interaction occurring between the second phenyl fragment and Ile433. Compound **10l** (carbon atoms in orange color) has two 7-chloro-4-(pyrrolidin-1-yl)quinolinium) cationic heads, one of which is inserted very similarly to compound **4** (Fig. 3B): the 4-pyrrolidin fragment is inserted into the additional binding site and stabilized by hydrophobic interactions, while the 7-chloroquinolinium moiety is situated into the Cho binding site and stabilized by cation-π interactions. The second cationic head is also inserted into the protein, being stabilized by hydrophobic interactions with Ile433 and Arg117, and the phenyl group connected to this cationic head is also stabilized by cation-π interaction with Phe435. The most notable effect in these molecules is the conformation of the linker, since the 1,2-dioxoethane fragment adopts a synclinal conformation due to the gauche effect of the O-C-C-O bonds. This conformation of the linker allows the total insertion of compound **10l** inside the Cho binding site, and also favors the insertion of compound **10a**.

Figure S2 of Supplementary Information shows the resulting pose of compounds **10b**, **10d**, and **10e**. The pose of compound **10b** is very similar to that of compound **10a**, the pyridinium moiety being slightly more separated from Tyr333 due to the higher volume of the pyrrolidine fragment, but the interaction of the whole molecule with the Cho binding site is very similar to that of compound **10a**. Compound **10d** shows also a similar pose to that of compound **10a**, though slightly more inserted into the Cho binding site due to the smaller volume of the quinuclidine cationic head. The resulting pose of compound **10e** is also similar, being slightly outside compound **10a** due to the establishment of two *H*-bond between the 3-OH groups and Asn305 and Glu434, respectively. Figure S3 of Supplementary Information shows the resulting pose of compounds **10f-k** and **10c**. Compounds **10f-k** have shown a pose very similar to that of compound **10l** (Fig. 3D), one of the cationic heads being inserted inside the additional binding site and into the Cho binding site. The second cationic head is also inserted into the protein being stabilized by hydrophobic interactions with Arg117 and Ile433, and the phenyl group of this cationic head is also stabilized by π-cation interaction with Phe435. The resulting pose of compound **10c** shows a slight difference. This compound has two 4-((4-chlorophenyl)(methyl)amino)pyridinium cationic heads. One cationic head is also inserted into the additional binding site and into the choline binding site, as in compound **4**, and the second cationic head is also inserted into the enzyme, but with a different orientation. Nevertheless, the most noteworthy effect is that in the resulting pose of these compounds the 1,2-dioxoethane fragment also adopted a synclinal conformation and, for this reason, all these compounds should be completely inserted into the enzyme and probably will show good ChoKα1 inhibition.

### Inhibition of ChoKα1 enzymatic activity

It has been reported that a potent anticancer effect inducing maximal apoptosis is achieved only when ChoKα1 expression is specifically knocked down, without affecting ChoKβ levels^47^. Thus, in an initial step we evaluated whether these compounds have a selective behaviour on ChoKα1.

We selected the most representative compounds of each family, (**10a** for pyridinium compounds and **10f**, **10g**, **10k**, and **10l** for quinolinium compounds) and, since selectivity may be explained by a reduced flexibility of Trp353 in ChoKβ compared to its homologue Trp420 in ChoKα1, as was proposed for **HC-3**^31^, tryptophan fluorescence assays were performed (Figure S4 of Supplementary information). As expected from their chemical structures similar to that of compound **4**, the results of the spectroscopy analysis, depicted in Table 1, showed that the *K*_d_ values of these compounds for ChoKα1 were in the low μM range (0.241–0.700 μM), indicating high affinity for the enzyme. These results agree with the first experimental validation of the docking studies described above.

Table 2 summarizes the clog *P* calculated by Pallas (3.8.1.1. Prolog*P*) and the inhibitory effect on purified human ChoKα1 activity.

Of all tested compounds, the ones that present an alkylamine or a cycloalkylamine substituted at position 4 of the pyridinium or quinolinium system, **10a-b** and **10j-l**, offer the best results in terms of enzyme inhibition and antiproliferative assays.

Regarding ChoKα1 inhibitory effect, all compounds showed a micromolar activity, comparable to that of the two reference compounds **RSM932A** and **MN58b**. The docking studies indicated that all compounds could be inserted into the choline binding site and could be grouped into two families on the basis of their different insertion modes. Compounds **10a**, **10b**, **10d**, and **10e** could be inserted similarly to compound **2**. Compound **10a** showed good *Hs*ChoKα1 inhibition (IC_50_ = 1.0 μM), thanks to the presence of the 1-benzyl-4-(dimethylamino)pyridinium fragment, which performs a strong π-cation interaction with the Cho binding site (Fig. 3C), this having been described as one of the most efficient moieties for ChoKα1 inhibition^33^. Compound **10b** showed a reduced ChoKα1 inhibition (IC_50_ = 9.56 μM) attributable to the volume of the 4-(pyrrolidin-1-yl)pyridinium cationic head, causing a decrease in the π-cation interaction (Figure S2A). In this family, compound **10d** showed very poor ChoKα1 inhibition (IC_50_ = 37.54 μM). This result may be due to the presence of the quinuclidine and the consequently low lipophilicity (clog *P* = −1.01) cationic head that probably prevented the interactions with Tyr333, Tyr354, Trp420, and Tyr440 (Figure S2B). However, although compound **10e** had a 3-hydroxyquinuclidine cationic head and low lipophilicity (clog *P* = −2.42), it showed better ChoKα1 inhibition (IC_50_ = 9.51 μM) than could be explained by the establishment of two additional *H*-bonds with the enzyme (Figure S2C).

In summary, when compounds bind to the choline site, such as compound **2** (compound **10a-b, d-e**), low lipophilicity values are sufficient to achieve good inhibition of the enzyme, the pydidinic ring being the most appropriate moiety, and the quinuclidine ring decreases the activity unless the lack of aromatic ring is offset by the formation of a *H* bond provided by the hydroxyl group.

The second group interacted with the choline binding site as compound **4**. Compounds **10c 10j-l** showed a good ChoKα1 inhibition (IC_50_ = 1.63, 1.66, 2.02 and 0.92 μM respectively), while compounds **10f-i** showed a slightly reduced ChoKα1 inhibition (IC_50_ = 6.85, 3.27, 2.79 and 16.22 μM, respectively). All these compounds had a 4-substituted and 7-substituted quinolinium cationic head, **10f-l**, except **10c**, which had the rest of the *N*-methylanilino at the 4 position of the pyridinium cationic head. The structure of the 4-substituted fragment conditions the inhibitory activity of these molecules. In fact, compounds **10j-l** had a 4-cycloalkylamino fragment, while compounds **10c** and **10f-i** had a 4-*N*-methylanilino substituent. The resulting IC_50_ values indicated that the 4-cycloalkylamino favored the ChoKα1 inhibition, probably promoting a more effective insertion into the additional binding site. In this group the chloro atom increased the volume of molecule and the lipophilicity. Thus, the compound **10l** offered the right balance between volume and lipophilicity. The perhydroazepine group (**10i**) provided the volume to be inserted into the choline binding site properly and the addition of a choro atom slightly depressed the activity (**10j**).

Conversely, the compounds with an *N*-methylanilino system at the 4 position of the pyridinium or quinolinium cationic heads showed lower IC_50_ than did those in the alkylamine system. In these compounds, the chloro atom in *para* position of 4-(methyl(phenyl)amino)quinolinium or pyridinium fragment (**10c**, **10g** and **10i**) or in position 7 of the quinolinium ring (**10h** and **10i**) seemed to play an essential role in the enzyme inhibition. In fact, the presence of the chloro atom allowed the cationic head to be accommodated at the choline-binding site, likely by an increase in the lipophilicity provided by the halogen atom, regardless of where the halogen was located (**10c**, **10g**, and **10h** IC_50_ = 1.63, 3.27, and 2.79 μM, respectively). A direct correlation between volume-lipophilic activity was found in these compounds (**10c**, **10f-i**), so that the less bulky and lipophilic compounds offered the best values (**10c**, ChoKα1, IC_50_ = 1.66 μM, clog *P* = 1.8) while adding a second aromatic ring slightly diminished the activity of **10f-h**. On the other hand, the two chloro atoms present in compound **10i** made the molecule too bulky to bind to the choline site, lowering its inhibition activity (ChoKα1, IC_50_ = 16.22 μM), and raising its lipophilicity (clog *P* = 4.23). However, the absence of chloro atom in **10f** also considerably decreased ChoKα1 inhibition (IC_50_ = 6.85 μM), highlighting the important role of the chloro atom.

Regardless the binding mode to the enzyme, the best inhibitory activities were found when a fragment of alkylamine or cycloalkylamine was present at the 4 position in pyridinium or quinolinium cationic heads, such as **10a**, **10j**, **10k**, and **10l** (IC_50_ = 1.0, 1.66, 2.02, and 0.92 μM, respectively), These results reveal that the volume of the cationic head probably fits properly into the choline binding site. The only exception was **10b** (IC_50_ = 9.56 μM), and this was a consequence of the higher volume of the 4-(pyrrolidin-1-yl)pyridinium cationic head that diminished the π-cation interaction (Figure S2A) mentioned above.

### Cancer-cell growth inhibition

All compounds were evaluated for their antiproliferative activity against a panel of nine different human tumor-cell lines (Table 2). All were given GI_50_ values generally lower than 1 μM, some of them even at nanomolar concentrations. Only two compounds, i.e. **10d** and **10e**, registered GI_50_ values higher than 10 μM against all tested lines.

**Compounds 10a**, **10b, 10f**, and **10l** offered the best antiproliferative activities against all cell lines. In particular, **10a** gave GI_50_ values ranging from 0.027 to 0.12 μM, although the best value was by **10f** in MDA-MB-231 cell line (GI_50_ = 0.001 μM). Compared to quinolinium derivatives, in general the pyridinium moiety provided better results in all tested cell lines.

In the pyridinium family, the best results were found when the substitution was an alkyl or cycloalkylamine (**10a**-**b**). The switching of dimethylamine to pyrrolidine caused a curious decline in activity for the majority of cell lines (**10b**, GI_50_ = 0.042 μM to 4.5 μM). The replacement of this tertiary amine by a conjugate aromatic system (4-(4-chloro-*N*-methylanilino)pyridinium, **10c**) resulted in lower activity (GI_50_ = 2.1 μM to 7.3 μM). It bears noting that despite of their low lipophilicity (**10a**, clog *P* = −0.36 and **10b** clog *P* = 0.36), the compounds **10a-b** provided better results than did compound **10c** (clog *P* = 1.8). This finding can be explained by the different mode to bind to enzymes of these compounds, as mentioned above.

Replacing the pyridinium system with quinuclidine as cationic head, such as compounds **10d** and **10e**, led to a total loss of activity in all cell lines. This result may be due to the very low lipophilicity of **10d**-**e** (clog *P* = −1.01 and −2.42 respectively) and to the low IC_50_ value.

Regarding the quinolinium family (**10f-l**), all compounds had GI_50_ in the range of submicromolar values, **10l** being the best compound (0.007 μM for Jurkat cells). No differences in the activity were detected between the different substituents in the 4 position upon the quinolinium system, since **10f** and **10l** (with *N*-methylanilino and 4-pyrrolidine in the 4 position, respectively) offer the best antiproliferative activity over nearly all the cell lines. However, the presence or not of a chloro atom over the quinolinium system, seems to play a crucial role in inhibiting cell growth. The chloro atom provided not only a higher lipophilicity to these compounds, improving the passage through the plasma membrane, but also a larger volume that impaired the insertion in the choline binding site. In fact, compound **10f**, which has an *N*-methylanilino group at the 4 position without any chloro atom, exhibited better antiproliferative activity than did compound **10h** or **10g**, which have a chloro atom in the quinolinium.

However, **10f** registered a lower value of ChoKα1 inhibition (IC_50_ = 6.86 μM) than **10g** and **10h** (IC_50_ = 3.27 and 2.79 μM, respectively) with chloro in *para* position of the *N*-methylanilino system (**10g**) or in 7 position of quinolinium fragment (**10h**). The chloro atom provided lipophilicity and good results of enzyme inhibition but this lipophilicity can allow binding to other targets in the cancer cells. On the other hand, the presence of two chloro atoms make compound **10i** too bulky to inhibit the enzyme (IC_50_ = 16.22 μM), strongly reducing the antiproliferative activity, and it is also more lipophilic (clog *P* = 4.23), and thus does not bind to other targets. In conclusion, among the quinolinium family, the compounds with an *N*-methylanilino system upon the 4 position (**10f-i**), **10f** offers the best antiproliferative values but lower inhibition of the enzyme (6.85 μM). This could be due to a greater affinity of **10f** by the enzyme in a whole cancer cell while the higher lipophicility of **10g-h** could help them to be more suitable for binding to other targets. This highlights the need for a balance between lipophilicity, inhibitory activity of the enzyme isolated (affinity and selectivity), and antiproliferative activity for achieving successful results.

The insertion of chloro in position 7 as in compound **10k** improves the activity. This suggests that although the enzymatic inhibitory activity is almost the same for these compounds (IC_50_ from 0.92 to 2.02 μM), the chloro leads to an appreciable increase in the antiproliferative activity (**10j** vs. **10k**) in nearly all the cell lines.

Finally, **10a** and **10l** were the compounds with the best GI_50_ values in almost all the cell lines. The two belong to different subfamilies. The first one, **10a**, has a residue of dimethylaminopyridinium as a cationic head which could offer the best volume to fit in a choline binding site, while the compound **10l** has a residue of 7-Chloro-4-pyrrolidinequinolinium which provides more lipophilicity to the quinolinium moiety, allowing the compound to cross the plasma membrane more easily.

### Trypan blue exclusion assay

**Figure 4:**
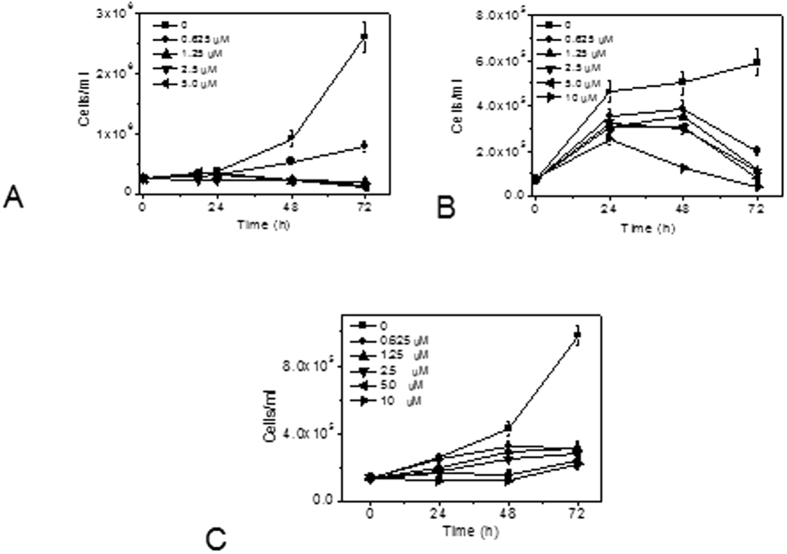
Cell viability evaluated by trypan blue count in Jurkat cells (**A**) HeLa cells (**B**) and MDA-MB-231 (**C**) after incubation with the indicated concentrations of compound **10a**. Data are presented as mean±SEM of three independent experiments.

The cell viability in the presence of **10a** was also evaluated by the trypan blue exclusion assay. The results depicted in Fig. 4 reflect that **10a** significantly inhibited cell growth in three cell lines (Fig. 4 panel A Jurkat, Panel B = HeLa; panel C = MDA-MB-231) tested in a concentration-dependent manner, roughly confirming the results found with the MTT test (see Table 2). Notably, we observed that the inhibition of cell proliferation was not dependent on the presence of the molecule in the incubation medium. In fact, experiments in which the cells treated with **10a** were harvested, washed, and incubated with fresh medium without **10a** proliferation continued to be inhibited, suggesting that the catalytic activity of the enzyme may have been irreversibly inhibited (Figure S5 of Supplementary information).

### Effect of 10a in non-tumoral cells

We investigated the effect of the most active compound (**10a**) in non-tumoral cells e.g. human lymphocytes and human umbilical-vein endothelial cells (HUVEC) from healthy donors. As shown in Table 3, in general, in resting lymphocytes, fibroblasts, and HUVEC, compound **10a** had lower toxicity compared to tumor cells. Instead, in lymphocytes stimulated with a mitogen (e.g. phytohematoagglutinin, Pha), the compound had cytotoxicity comparable to that seen in tumor cells, indicating a certain preference only towards proliferating cells. In this context, it bears noting that other choline kinase inhibitors such as **MN58b** or **RSM932A** presented low or reduced cytotoxicity in oncogene-transformed cells and in tumor cells, in agreement with previously published data^44^,^48^.

### The crystal structure of the complex ChoKα1/10a shows that the compound binds to the choline binding site

**Figure 5:**
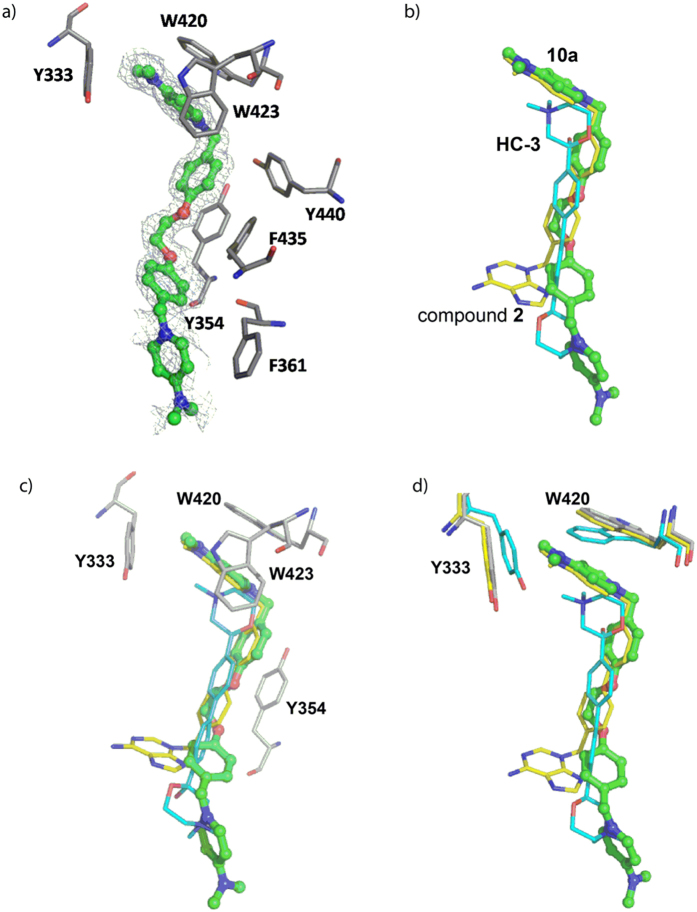
(**a**) Active site of *ChoK*α1 in complex with compound **10a**. Unbiased difference electron density maps are shown at 2.2 σ. **(b**) Superimposition of **10a** with **HC-3** (PDB ID: 3G15) and compound **2** (PDB ID: 3ZM9). (**c**) Residues that stabilize the positive charge of the cationic head in the three superimposed ligands. (**d**) Residues that undergo the most notable conformational changes when compounds **2** or **10a** bind to the choline binding site.

Although the docking studies clearly suggested that the compounds bind to the choline binding site (Fig. 3, Figures S2 and S3 in the Supplementary information), we carried out further crystallization experiments with the most active ChoKα1 inhibitors, **10a** and **10l**, in order to compile more consistent data concerning their binding mode (Fig. 5).

Despite the large number of trials, we managed to solve only the crystal structure of ChoKα1 in complex with compound **10a** at 1.45 Å (see Methods and Supplementary information, Table S2). The other compound (**10l**) was too hydrophobic (clog *P* = 2.36, Table 2) and therefore insoluble in the mother liquor.

For ChoKα1/**10a** complex, successive iterative model building and refinement cycles were carried out to produce a final model with good refinement statistics (R = 0.197, R_free_ = 0.218, Table S2).

The structure is a monomer formed by a small *N*-terminal and a large *C*-terminal domain. Whereas the ATP binding site is located in a cleft formed by *N*- and *C*-terminal domains, the choline binding site is found in a deep hydrophobic pocket. One molecule of compound **10a** was visualized at the choline binding site with good electron density (Fig. 5a). One 1-benzyl-4-(dimethylamino)pyridinium fragment was deeply positioned within the pocket and established π-cation interactions with Trp423 and Trp420, whereas it set π-π interactions with Tyr333, Tyr354, Phe435, and Tyr440 (Fig. 5a). The second 1-benzyl-4-(dimethylamino)pyridinium moiety was directed towards the outside part of the choline binding site, where it established π-π and hydrophobic interactions with residues Phe361 and Ile433, respectively (Fig. 5a).

When the crystal pose was compared with the docked one, few differences were found between the two, especially regarding the conformation that the first 1-benzyl-4-(dimethylamino)pyridinium fragment adopted (Supporting information, Figure S6).

This moiety was completely superimposed in the two poses, indicating the accuracy of the theoretical predictions. Nevertheless, the second 4-(dimethylamino)pyridinium moiety was flipped almost 90° towards the residue Phe361 but not towards the residue Ile433, as the docking had predicted. The reason for this difference is that the pyridinium ring set π-π interactions with Phe361 in the crystal pose, increasing the stability of the ligand-protein complex.

Remarkably, the positive charge of the quaternary ammonium of the first 1-benzyl-4-(dimethylamino)pyridinium fragment was positioned at the choline binding site in the same place as one of the previously reported ChoKα1 inhibitors, such as compound **2** (PDB ID:4BR3) and **HC-3** (PDB ID: 3G15) (Fig. 5b). This indicates that common residues should participate in the positive-charge stabilization regardless of the chemical nature of the spacer groups. As reflected in Fig. 5c, these residues are Tyr333, Tyr354, Trp420, and Trp423, which set parallel π-cation and π-π interactions with the quaternary ammonium and the aromatic rings of the 1-benzyl-4-(dimethylamino)pyridinium fragment. Nevertheless, depending on the spacer and the cationic head of the compounds, some conformational changes in some of these residues were observed. For instance, residues Trp420 and Tyr333 underwent a noticeable retraction to open the hydrophobic cavity when compound **10a** or compound **2** bound the protein in order to accommodate the positive charge of the cationic head (Fig. 5d).

### 10a and G1 phase cell-cycle arrest

**Figure 6:**
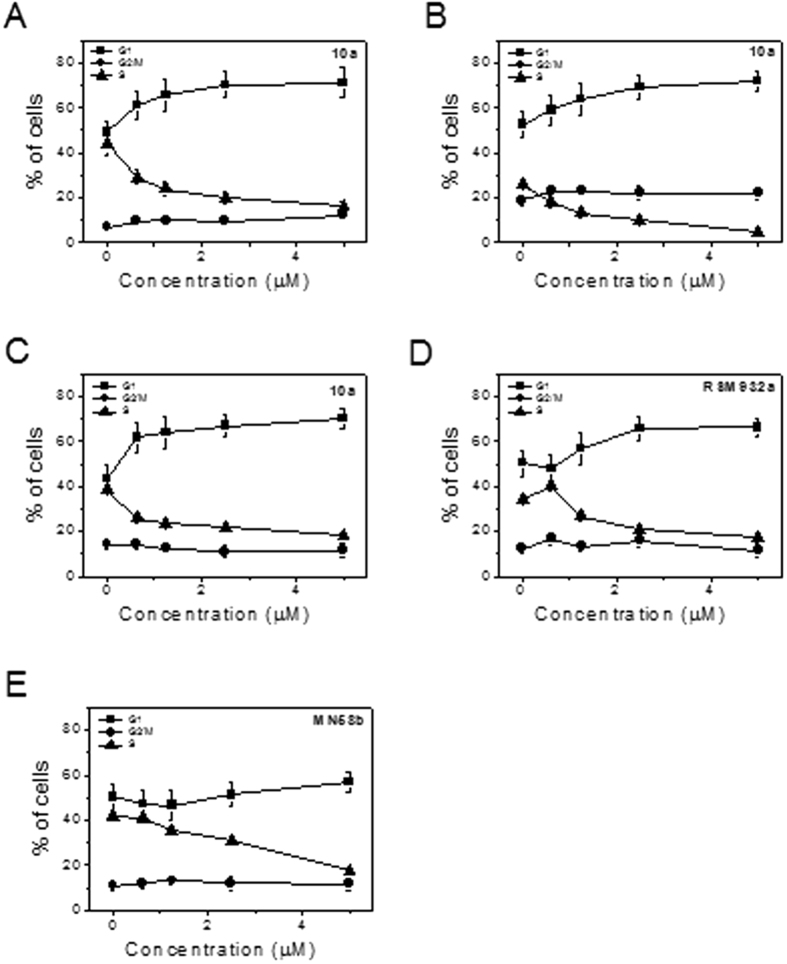
Percentage of cells in each phase of the cell cycle in Jurkat (Panels **A**), MCF-7 (Panel **B**) and MDA-MB231 cells (Panels **C–E**) treated with the indicated compounds at the indicated concentrations for 24 h. Cells were fixed and labeled with PI and analyzed by flow cytometry as described in the experimental section.

Compound **10a** induced a G1 arrest of the cell cycle, which occurred in a concentration-dependent manner in the three cell lines tested (Jurkat, MCF-7 and MDA-MB-231). Together with the G1 increase, a concomitant reduction was found in the S phase (Fig. 6). Notably, cells with hypodiploid DNA content, suggestive of activation of apoptotic signaling, were not detected (data not shown). Similar results were also found with the two lead compounds **RSM932A** and **MN58b**, suggesting a common mechanism of action. Our results agree with the data of Granata *et al.*^16^ which showed that ChoKα downregulation in ovary-cancer cells inhibits cell proliferation without affecting survival signaling pathways whereas, a reduction in the S-phase, proportional to growth inhibition, was observed in cells knocked down for ChoKα1. The cell cycle also showed a slight G1 cell-cycle arrest in silenced cells compared with controls. On the contrary, Sanchez-Lopez *et al.*^15^ showed that **RSM932A** and **MN58b** induce a significant decrease of the G1 phase in breast- and colon-cancer cells without any alteration of the other phases of the cell cycle. It should be noted that these data correspond to a concentration (15 μM) higher than that used in the present study.

### 10a induces low levels of apoptosi**s**

To evaluate whether **10a** causes cell death, we conducted a biparametric cytofluorimetric analysis using PI, which stains DNA and enters only dead cells, together with fluorescent immunolabeling of the protein annexin-V, which binds to PS in a highly selective manner. Dual staining for annexin-V and with PI enables the discrimination between live cells (annexin-V^−^/PI^−^), early apoptotic cells (annexin-V^+^/PI^−^), late apoptotic cells (annexin-V^+^/PI^+^), and necrotic cells (annexin-V^−^/PI^+^). For a positive control, we used two well-known anticancer drugs such as Cis-Pt and Etoposide that in all cell lines tested induce significant apoptosis.

**Figure 7:**
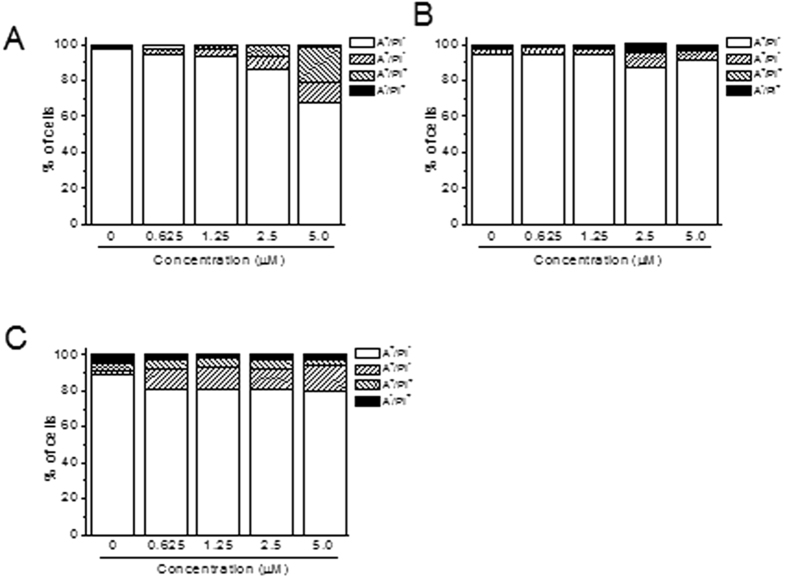
Flow cytometric analysis of apoptotic cells after treatment of Jurkat (panel **A**), MCF-7 (panel **B**) and MDA-MB-231 (panel **C**) cells with **10a** at the indicated concentrations after incubation for 72 h. The cells were harvested and labeled with annexin-V-FITC and PI and analyzed by flow cytometry. As positive controls Cis-Pt and Etoposide (Eto) were used at the concentration of 50 μM and 5 μM, respectively. Data are represented as mean ±SEM of three independent experiments.

As depicted in Fig. 7, **10a** after 72 h of incubation induced a modest increment in apoptotic cells only in Jurkat cells while both in MCF-7 and MDA-MB-231 the increase appeared not to be significantly different from that of the untreated cells.

## Conclusions

In conclusion, the synthesis of a novel family of 1,1′-(((ethane-1,2-diylbis(oxy))bis(4,1-phenylene))bis(methylene))-bispyridinium or –bisquinolinium bromide (**10a-l**) and their evaluation as inhibitors of choline kinase against a panel of cancer-cell lines are described. These compounds were efficiently synthesized in three steps, starting from the building block **6.** The chemistry used was appropriate to obtain the designed target compounds, and both the yield and the time of reaction were improved considerably with microwave irradiation.

Preliminary docking studies performed on both crystal structures, ChoKα1/**2** (PDB ID: 4BR3) and (ChoKα1/**4** PDB ID: 4CG8), and the analysis of the resulting poses, indicated that these compounds (**10a-l**) could adopt similar behaviour to that of compound **2** or to compound **4** thanks to the synclinal conformation of the linker that allowed the insertion of these molecules inside the Cho binding site and consequently enhanced the antiproliferative effect. The first experimental validation of the docking studies is shown with the results of tryptophan assays for these compounds, which offer very good Kd values. Among all the compounds, those belonging to the families of pyridinium and quinolinium offered similar or better IC_50_ ChoKα1 than did the lead compounds **MN58b** and **RSM932A**. In fact, the best inhibitors were **10a** and **10l**, and the crystal structure of ChoKα1/**10a** showed that the compound binds to the choline binding site, indicating the accuracy of the theoretical predictions, wherein the first one-benzyl- 4- (dimethylamino) pyridinium represents the appropriate fragment to inhibit the enzyme^33^.

Also, we have shown these compounds to have an excellent antiproliferative profile, better than that of the two lead compounds **RSM932A** and **MN58b** in a panel of human tumor-cell lines. More importantly, our compounds presented lower or reduced toxicity in some non-tumor-cell lines in comparison to transformed cells. Indeed our results agree with previous observations indicating increased ChoKα1 activity in cell cultures treated with growth factors or insulin^23^,^49^,^50^.

In this context it is important to note that our results indeed confirm these previous findings, and compound **10a** in fact presented higher activity only in rapidly proliferating cells such as mitogen-stimulated lymphocytes in comparison to quiescent cells.

Another important finding is that **10a** significantly arrested the cell cycle in G1 together with a sharp reduction of the S phase, confirming their ability to inhibit cell growth. Curiously, despite their ability to block cell proliferation, **10a** induced a low proportion of cell death, as reflected by a low level of Annexin-V positive cells (Fig. 7). Notably, this occurred also for the two reference compounds **RSM932A** and **MN58b**, which even at the maximum concentration used showed a negligible percentage of apoptotic cells, according to the analysis of the cell cycle. It is important to note that, although some reports^26^ indicate these two compounds may induce apoptosis, this takes place at concentrations much higher than the IC_50_ (15 μM), which can have an off-target effect.

This intriguing aspect is under active investigation by our group. Nevertheless, our data demonstrate that **10a** is a highly promising new Chokα1 inhibitor and is worthy of further preclinical evaluation as a potential anticancer drug.

## Methods

### Chemistry

#### General procedure C for the synthesis of the final compounds 10a-l

A solution of 1eq 1,2-bis(4-bromomethylphenoy)ethane (**8**) in dry CH_3_CN was added drop by drop to a solution of **9a-l** (2 eq) in dry CH_3_CN under argon conditions. The mixture was heated under reflux for a 3 additional days and, after cooling down to room temperature, washed with diethyl ether and hexane, filtered, and dried under vacuum to afford **10a-l** as a solid product.

### Characterization data for final products are described below

#### 1,1′-(((ethane-1,2-diylbis(oxy))bis(4,1-phenylene))bis(methylene))bis(4-(dimethylamino)pyridinium) bromide (10a)

Following general procedure C furnished **10a** as a yellow solid, yield 42%, mp: 62–63 °C. ^1^H NMR (300 MHz, CD_3_OD) *δ*: 8.20 (d, *J* = 7.86 Hz, 4H), 7.35 (d, *J* = 8.73 Hz, 4H), 7.02 (d, *J* = 8.73 Hz, 4H), 6.99 (d, *J* = 7.86 Hz, 4H), 5.30 (s, 4H), 4.33 (s, 4H), 3.24 (s, 12H). ^**13**^**C RMN** (75 MHz, CD_3_OD) *δ*: 161.80 × 2, 158.87 × 2, 143.76 × 4, 131.98 × 4, 129.20 × 2, 117.30 × 4, 109.93 × 4, 68.86 × 2, 62.22 × 2, 41.20 × 4. **HRMS** (m/z): [M]^2+^ calcd for C_30_H_36_N_4_O_2_: 242.1419, found: 242.1409.

#### 1,1′-(((ethane-1,2-diylbis(oxy))bis(4,1-phenylene))bis(methylene))bis(4-(pyrrolidin-1-yl)pyridinium) bromide (10b)

Following general procedure C furnished **10b** as a brown solid, yield 48%, mp: 129–130 °C. ^1^H NMR (300 MHz, CD_3_OD) *δ*: 8.17 (d, *J* = 7.77 Hz, 4H), 7.34 (d, *J* = 8.73 Hz, 4H), 7.01 (d, *J* = 8.73 Hz, 4H), 6.84 (d, *J* = 7.77 Hz, 4H), 5.28 (s, 4H), 4.32 (s, 4H), 3.54 (t, *J* = 6.84 Hz, 8H), 2.11 (t, *J* = 6.86 Hz, 8H), ^13^C NMR (75 MHz, CD_3_OD) *δ*: 161.79 × 2, 156.05 × 2, 143.68 × 4, 131.92 × 4, 129.31 × 2, 117.29 × 4, 110.51 × 4, 68.86 × 2, 62.26 × 2, 50.28 × 4, 26.98 × 4. HRMS (m/z): [M]^2+^ calcd for C_34_H_40_N_4_O_2_: 268.1576, found: 268.1568.

#### 1,1′-(((ethane-1,2-diylbis(oxy))bis(4,1-phenylene))bis(methylene))bis(4-((4-chlorophenyl)(methyl)amino)pyridinium) bromide (10c)

Following general procedure C furnished **10c** as a white solid, yield 30%, mp: >300 °C. ^1^H NMR (600 MHz, CD_3_OD) *δ*: 8.28 (d, *J* = 8.5 Hz, 4H), 7.58 (d, *J* = 8.5 Hz, 4H), 7.37 (m, 8H), 7.02 (d, *J* = 8.4 Hz, 4H), 6.92 (m, 4H), 5.35 (s, 4H), 4.33 (s, 4H), 3.53 (s, 6H). ^13^C NMR (75 MHz, CD_3_OD) *δ:* 160.85 × 2, 158. 31 × 2 143.52 × 4, 135.52 × 2, 131.94 × 4, 131.13 × 4, 129.28 × 4, 127.95 × 2, 116.27 × 8, 110.27 × 2, 67.81 × 2, 61.64 × 2, 30.55 × 2. **HRMS** (m/z): [M]^2+^ Calcd for C_20_H_19_N_2_OCl: 338.1186, found: 338.1194.

#### 1,1′-(((ethane-1,2-diylbis(oxy))bis(4,1-phenylene))bis(methylene))bis(quinuclidinium) bromide (10d)

Following general procedure C furnished **10d** as a white solid, yield 56%, mp: >300 °C. ^1^H NMR (300 MHz, CD_3_OD) *δ*: 7.47 (d, *J* = 8.78 Hz, 4H), 7.13 (d, *J* = 8.78 Hz, 4H), 4.43 (s, 4H), 4.35 (s, 4H), 3.48 (m, 12H), 2.18 (dt, *J* = 6.44, 3.23 Hz, 2H), 2.01 (dt, *J* = 8.23, 3.23 Hz, 12H). ^13^C NMR (75 MHz, CD_3_OD) *δ*: 162.80 × 2, 136.48 × 4, 121.54 × 2, 117.14 × 4, 69.52 × 2, 68.87 × 2, 56.39 × 6, 25.81 × 6, 22.33 × 2. HRMS (m/z) [M]^2+^ calcd for C_30_H_42_N_2_O_2_: 231.1623, found: 231.1628.

#### 1,1′-(((ethane-1,2-diylbis(oxy))bis(4,1-phenylene))bis(methylene))bis(3-hydroxyquinuclidinium) bromide (10e)

Following general procedure C furnished **10e** as a white solid, yield 63%, mp: 268–270 °C. ^1^H NMR (300 MHz, DMSO-*d*^6^) *δ*: 7.48 (d, *J* = 8.66 Hz, 8H), 7.13 (d, *J* = 8.66 Hz, 8H), 4.43 (s, 8H), 4.41 (s, 8H), 4.08, 3.64, 3.35, 3.04, 2.27, 2.10 (6m, 52H), ^13^C NMR (75 MHz, DMSO-*d*^6^) *δ*: 159.52 × 4, 134.43 × 8, 119.63 × 4, 114.77 × 8, 65.61 × 4, 62.26 66.37, 63.27, 53.46, 52.52, 26.83, 20.87, 17.29 × 4. HRMS (m/z) [M]^2+^ calcd for C_30_H_42_N_2_O_4_: 247.1572, found: 247.1565.

#### 1,1′-(((ethane-1,2-diylbis(oxy))bis(4,1-phenylene))bis(methylene))bis(4-(methyl(phenyl)amino)quinolinium) bromide (10f)

Following general procedure C furnished **10f** as a yellow solid, yield 64%, mp: 169–170 °C. ^1^H NMR (300 MHz, CD_3_OD) *δ*: 8.86 (d, *J* = 7.44 Hz, 2H), 8.13 (d, *J* = 8.37 Hz). 7.81 (dt, *J* = 5.57, 1.43 Hz, 2H), 7.62 (dd, *J* = 8.8, 1.3 Hz, 2H), 7.53 (m, 4H), 7.46 (t, *J* = 7.36 Hz, 2H), 7.40–7.29 (m, 12H), 7.03 (d, *J* = 8.76 Hz, 4H), 5.89 (s, 4H), 4.33 (s, 4H), 3.84 (s, 6H). ^13^C NMR (75 MHz, CD_3_OD) *δ*: 161.47 × 2, 160.80 × 2, 150.14 × 2, 148.54 × 2, 141.68 × 2, 135.51 × 2, 132.75 × 4, 130.63 × 4, 130.28 × 2, 130.15 × 2, 128.64 × 2, 127.85 × 2, 127.73 × 4, 122.29 × 2, 120.97 × 2, 117.30 × 4, 107.73 × 2, 68.85 × 2, 59.94 × 2, 46.75 × 2. HRMS (m/z) [M]^2+^ calcd for C_48_H_44_N_4_O_2_: 354.1732, found: 354.1736.

#### 1,1′-(((ethane-1,2-diylbis(oxy))bis(4,1-phenylene))bis(methylene))bis(4-((4-chlorophenyl)(methyl)amino)quinolinium) bromide (10g)

Following general procedure C furnished **10g** as a yellow solid, yield 70%, mp: 178–180 °C. ^1^H NMR (400 MHz, CD_3_OD) *δ*: 8.90 (d, *J* = 7.40 Hz, 2H), 8.16 (dd, *J* = 8.9, 0.6 Hz, 2H), 7.84 (dt, *J* = 5.64, 1.36 Hz, 2H), 7.67 (dd, *J* = 8.8, 1.2 Hz, 2H), 7.52 (d, *J* = 8.91 Hz, 4H), 7.41–7.37 (m, 8H), 7.33 (d, *J* = 8.77 Hz, 4H), 7.03 (d, *J* = 8.77 Hz, 4H), 5.91 (s, 4H), 4.33 (s, 4H). 3.82 (s, 6H), ^13^C NMR (75 MHz, CD_3_OD) *δ*: 161.48 × 2, 160.92 × 2, 148.81 × 2, 148.77 × 2, 141.68 × 2, 135.68 × 2, 135.58 × 2, 132.72 × 4, 130.69 × 4, 130.00 × 2, 129.28 × 4, 128.52 × 2, 128.20 × 2, 122.41 × 2, 121.13 × 2, 117.30 × 4, 108.36 × 2, 68.84 × 2, 60.09 × 2, 46.53 × 2. HRMS (m/z) [M]^2+^ calcd for C_48_H_42_N_4_O_2_Cl_2_: 388.1342, found: 388.1338.

#### 1,1′-(((ethane-1,2-diylbis(oxy))bis(4,1-phenylene))bis(methylene))bis(7-chloro-4-(methyl(phenyl)amino)quinolinium) bromide (10h)

Following general procedure C furnished the crude residue which was purified by flash chromatography using CH_2_Cl_2_: MeOH (9:1 v/v) as eluent to obtain **10h** as a yellow-green solid, yield 61%, mp: 181–183 °C. ^1^H NMR (300 MHz, CD_3_OD) *δ*: 8.82 (d, *J* = 7.50 Hz, 2H), 8.15 (d, *J* = 1.89 Hz, 2H), 7.56–7.53 (m, 6H), 7.47 (t, *J* = 7.4 Hz, 2H), 7.41–7.40 (d, *J* = 7.48 Hz, 4H), 7.34–7.31 (m, 6H), 7.29 (dd, *J* = 9.3, 1.9 Hz, 2H), 7.06–7.03 (d, *J* = 8.70 Hz, 4H), 5.85 (s, 4H), 4.34 (s, 4H), 3.82 (s, 6H, CH_3_). ^13^C NMR (75 MHz, CD_3_OD) *δ*: 161.58 × 2, 160.51 × 2, 149.76 × 2, 148.95 × 2, 142.51 × 2, 141.90 × 2, 132.92 × 4, 131.80 × 2, 130.77 × 4, 130.59 × 2, 128.35 × 2, 128.24 × 2, 127.72 × 4, 128.24 × 2, 120.68 × 2, 120.45 × 2, 117.41 × 4, 107.93 × 2, 68.86 × 2, 59.91 × 2, 46.87 × 2. HRMS (m/z): [M]^2+^ calcd for C_48_H_42_N_4_O_2_Cl_2_: 388.1342, found: 388.1331.

#### 1,1′-(((ethane-1,2-diylbis(oxy))bis(4,1-phenylene))bis(methylene))bis(7-chloro-4-((4-chlorophenyl)(methyl)amino)quinolinium) bromide (10i)

Following general procedure C furnished the crude residue which was purified by flash chromatography using CH_2_Cl_2_: MeOH (9:1 v/v) as eluent to obtain **10i** as a yellow solid, yield 39%, mp: 185–186 °C, ^1^H NMR (300 MHz, CD_3_OD) *δ*: 8.87 (d, *J* = 7.46 Hz, 2H), 8.20 (d, *J* = 1.93 Hz, 2H), 7.63 (d, *J* = 9.31 Hz, 2H), 7.55 (d, *J* = 8.78 Hz, 4H), 7.42 (d, *J* = 8.78 Hz, 4H), 7.40 (d, *J* = 1.96 Hz, 2H), 7.38 (d, *J* = 7.47 Hz, 2H), 7.35 (d, *J* = 8.73 Hz, 4H), 7.06 (d, *J* = 8.73 Hz, 4H, H-2), 5.88 (s, 4H), 4.35 (s, 4H), 3.82 (s, 6H, CH_3_). ^13^C NMR (75 MHz, CD_3_OD) *δ*: 161.59 × 2, 160.65 × 2, 149.22 × 2, 148.45 × 2, 142.50 × 2, 142.06 × 2, 135.92 × 2, 132.88 × 4, 131.68 × 2, 130.85 × 4, 129.33 × 4, 128.71 × 2, 128.15 × 2, 120.82 × 2, 120.61 × 2, 117.40 × 4, 108.57 × 2, 68.85 × 2, 60.05 × 2, 46.69 × 2. HRMS (m/z) [M]^2+^ calcd for C_48_H_40_N_4_O_2_Cl_4_: 422.0953, found: 422.0952.

#### 1,1′-(((ethane-1,2-diylbis(oxy))bis(4,1-phenylene))bis(methylene))bis(4-(azepan-1-yl)quinolinium) bromide (10j)

Following general procedure C furnished **10j** as a yellow solid, yield 67%, mp: 75–77 °C. ^1^H RMN (300 MHz, CD_3_OD) *δ*: 8.54 (d, *J* = 7.73 Hz, 2H), 8.42 (dd, *J* = 8.62, 1.28 Hz, 2H), 8.05 (dd, *J* = 8.83, 1.05 Hz, 2H), 7.90 (dt, *J* = 5,67, 1,33 Hz, 2H), 7.66 (dt, *J* = 5.77, 1.15 Hz, 2H), 7.27 (d, *J* = 8.80 Hz, 4H), 7.10 (d, *J* = 7.74 Hz), 7.00 (d, *J* = 8.80 Hz, 4H), 5.74 (s, 4H), 4.30 (s, 4H), 4.09 (m, 8H), 2.09 (m, 8H), 1.75 (dt, *J* = 5.40, 2.54 Hz, 8H), ^13^C NMR (75 MHz, CD_3_OD) *δ*: 161.91 × 2, 161.34 × 2, 146.48 × 2, 142.10 × 2, 135.52 × 2, 130.48 × 4, 130.44 × 2, 128.98 × 2, 127.04 × 2, 121.41 × 2, 120.33 × 2, 117.22 × 4, 104.55 × 2, 68.84 × 2, 58.99 × 2, 56.06 × 4, 29.37 × 4, 29.23 × 4. HRMS (m/z) [M]^2+^ calcd for C_46_H_52_N_4_O_2_: 346.6700, found 346.2039.

#### 1,1′-(((ethane-1,2-diylbis(oxy))bis(4,1-phenylene))bis(methylene))bis(4-(azepan-1-yl)-7-chloroquinolinium) bromide (10k)

Following general procedure C furnished the crude residue which was purified by flash chromatography using CH_2_Cl_2_: MeOH (8:1 v/v) as eluent to obtain **10k** as a white solid, yield 41%, mp: 87–88 °C. ^1^H RMN (400 MHz, CD_3_OD) *δ*: 8.51 (d, *J* = 7.75 Hz, 2H), 8.40 (d, *J* = 9.21 Hz, 2H), 8.07 (d, *J* = 1.82 Hz, 2H), 7.29 (d, *J* = 8.64 Hz, 4H), 7.65 (dd, *J* = 9.2, 1.9 Hz, 2H), 7.11 (d, *J* = 7.78 Hz, 2H), 7.03 (d, *J* = 8.64 Hz, 4H), 5.72 (s, 4H), 4.33 (s, 4H), 4.08 (m, 8H), 2.08 (m, 8H), 1.74 (m, 8H). ^13^C NMR (75 MHz, CD_3_OD) *δ* :161.56 × 2, 161.44 × 2, 146.77 × 2, 142.95 × 2, 141.82 × 2, 132.31 × 2, 130.59 × 4, 128.57 × 2, 127.45 × 2, 119.88 × 2, 119.78 × 2, 117.32 × 4, 105.00 × 2, 68.85 × 2, 59.00 × 2, 56.12 × 4, 29.28 × 4, 29.16 × 4. HRMS (m/z) [M-Br]^+^ calcd for C_46_H_50_N_4_O_2_Cl_2_Br : 839.24949, found: 839.2494.

#### 1,1′-(((ethane-1,2-diylbis(oxy))bis(4,1-phenylene))bis(methylene))bis(7-chloro-4-(pyrrolidin-1-yl)quinolinium) bromide (10l)

Following general procedure C furnished the crude residue which was purified by flash chromatography using CH_2_Cl_2_: MeOH (9:1 v/v) as eluent to obtain **10l** as a white solid, yield 48%, mp: 118–120 °C. ^1^H RMN (300 MHz, CD_3_OD) *δ*: 8.63 (d, *J* = 9.23 Hz, 2H), 8.51 (d, *J* = 7.69 Hz, 2H), 8.04 (d, *J* = 2.02 Hz, 2H), 7.66 (dd, *J* = 9.2, 2.0 Hz, 2H), 7.27 (d, *J* = 8.75 Hz, 4H), 7.01 (d, *J* = 8.75 Hz, 4H), 6.90 (d, *J* = 7.69 Hz, 2H), 5.71 (s, 4H), 4.32 (s, 4H), 4.02 (m, 8H), 2.20 (m, 8H). ^13^C NMR (75 MHz, CD_3_OD) *δ*: 161.37 × 2, 157.92 × 2, 146.91 × 2, 142.45 × 2, 141.76 × 2, 131.76 × 2, 130.45 × 4, 128.68 × 2, 127.71 × 2, 120.16 × 2, 119.73 × 2, 117.29 × 4, 104.64 × 2, 68.83 × 2, 59.06 × 2, 55.43 × 4, 24.64 × 4. HRMS (m/z) [M]^2+^ calcd for C_42_H_42_N_4_O_2_Cl_2_: 352.1337, found: 352.1353.

### Docking Studies

Molecular-modeling studies were performed by using Sybyl program^51^. Crystal structures of human ChoKα1 in complex with compounds **2** (PDB entry 4BR3) and **4** (PDB entry 4CG8) were used for docking studies. In both cases, using the Structure Preparation Tool module of Sybyl refined protein structure. Missing side chains of those residues situated far away from the binding sites were added and protein N-terminal and C-terminal were fixed with ACE and NME, respectively. Hydrogens and charges were also added and protonation type of Glu, Asp, Gln and Asp was analyzed and fixed. Hydrogen orientations were also checked in order to maintain intramolecular hydrogen bonds within the protein. Finally, the molecules of compounds **2** and **4** inserted into the ATP binding site were carefully checked to assure the correction of these molecules. Structures of compounds **10a-l** were constructed from standard fragments of the Libraries of the Sybyl program, and used as ligands for docking studies. As previously described^52^, a new type of atom was necessary to define in order to build the molecules: N.ar4, the quaternary nitrogen of the pyridinium fragments. Additional parameters were also developed from initio calculations to optimize the geometry of these molecules Atomic charges were calculated by means of Gaussian Program^53^ and optimizations were undertaken using the BFGS method.

The Surflex-Dock^54^, module implemented in the Sybyl program was used for docking studies. Surflex Dock Protomol was prepared using compound **2** or **4** inserted into the ChoK binding site, with a threshold value of 0.5 and a Bloat of 0 A. Surflex-Dock GeomX (SFXC) protocol was used, the search grid was expanded in 5 Å, 50 additional starting conformations were used for each molecule and 30 conformations per fragment. The results were analyzed using the Sybyl program and the most stable pose for each molecule was chosen as the preferred one inside the ChoK enzyme. Figures were built using the PyMOL program^55^.

## Biological experiments

### Materials and Methods

[methyl-^14^C]choline chloride (55 mCi/mmol) was supplied by Perkin Elmer (Massachusetts, USA). Fetal bovine serum (FBS) was from The Cell Culture Company (Pasching, Austria). Minimal essential medium (MEM) was from Sigma-Aldrich (Madrid, Spain). Cell proliferation reagent WST-1 was from Roche Applied Sciences (Mannhein, Germany). Thin-layer chromatography (TLC) plates of Silica Gel 60 A were acquired from Analtech (Newark, DE, USA). Microwell plates and culture dishes were obtained from NuncTM (Langenselbold, Germany). All other reagents were of analytical grade.

### Determination of Human choline kinase α1 (ChoKα1) activity

The effect of the different inhibitors on human choline kinase (ChoK) was assayed in ChoKα1 purified as previously described^32^. In each experiment DMSO-assays were always run in parallel as a control. DMSO in no case exceeded a concentration of 0.1% in order to avoid unspecific ChoK inhibition. ChoK activity was assayed by measuring the rate of incorporation of ^14^C from [methyl-^14^C]choline into phosphocholine both in the presence or absence of different inhibitor concentrations. Briefly, the final reaction mixture contained 100 mM Tris (pH 8.5), 10 mM MgCl_2_, and 10 mM ATP, and 20 ng of purified ChoKα1. After the samples were preincubated at 37 °C for 5 min, the reaction was initiated with 1 mM [methyl-^14^C]choline (4500 dpm/nmol) and incubated at 37 °C for 10 min, the final volume being 55 μl. The assay was stopped by immersing the reaction tubes in boiling water for 3 min. Aliquots of the reaction mixture were applied to the origin of Silica Gel plates in the presence of phosphocholine (0.1 mg) and choline (0.1 mg) as carriers. The chromatography was developed in methanol/0.6% NaCl/28% NH_4_OH in water (50:50:5, v/v/v) as solvent. Phosphocholine was visualized under exposure to iodine vapor and the corresponding spot was scraped and transferred to scintillation vials for measurement of radioactivity by a Beckman 6000-TA (Madrid, Spain) liquid-scintillation counter. At least three experiments were performed in all assays. The 50% inhibitory concentrations (IC_50_ values) were determined from the % activity of the enzymes at different concentrations of synthetic inhibitors by using a sigmoidal dose-response curve (the ED_50_plus v1.0 software).

### Cloning and purification of ChoKα1

Details on cloning and purification of human *ChoK*α1 have been previously reported^32^.

### Tryptophan fluorescence quenching

All compounds were prepared in 100% DMSO. Their *K*_d_s against human ChoKα1 were measured by monitoring the quenching of tryptophan fluorescence. All experiments were performed in a Cary Eclipse spectrofluorometer (Varian) at 25 °C with the enzymes at 1 μM, and concentrations of compounds varying from 0.1 to 5 μM for ChoKα1 in 25 mM Tris, 150 mM NaCl, pH 7.5. Fluorescence emission spectra were recorded in the 300–400 nm range with an excitation wavelength of 280 nm, with slit width of 5 nm. Controls were determined by incubating the enzymes with equivalent amounts of DMSO. As indicated previously, data analysis was performed in Prism (GraphPad software) considering a model with a single binding site (Eq. 1), where **F0** is the intrinsic fluorescence of the enzyme in the absence of quencher (Q), **F**1 is the observed fluorescence at a given quencher concentration, **f**_a_ is the fractional degree of fluorescence, and **K**_d_ is the dissociation constant.


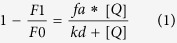


### Protein crystallography

ChoKα1 at 20 mg/ml in buffer 25 mM Tris/HCl, 150 mM NaCl pH 7.5 was used as the protein solution. The sitting-drop vapor-diffusion method was used to produce apo-crystals by mixing 0.5 μl of the protein solution and an equal volume of mother liquor (crystals appeared in 20% polyethylene glycol [PEG] 3350 and 0.25 M potassium isothiocyanate). Tetragonal crystals (space group P43212) grew within in 3–4 days and were soaked in 2 μL of the mother liquor with 0.2 μL of a dilution 500 mM of compounds **10a**, **10g**, **10h**, **10k**, and **10l** in DMSO (DMSO was at 10% final concentration in the mix whereas compounds were at 50 mM) for two days. Only crystals soaked with compound **10a** contained the compound. The crystals used in this study were cryoprotected in mother-liquor solutions containing 20% ethylenglycol and frozen in a nitrogen gas stream cooled to 100 K.

Diffraction data of the binary complexes were collected at beamline I04-1 (Diamond, Oxford). The data was processed and scaled using the XDS package and CCP4 software, relevant statistics are given in Table S2 of Supplementary information

### Structure determination and refinement

The structure of the binary complex was solved by molecular replacement using PDB ID 3G15 as a template. Initial phases were further improved by cycles of manual model building in Coot and refinement with REFMAC5. The final model was validated with PROCHECK, model statistics are given in Table S2 of the supplemental information section. Coordinates and structure factors have been deposited in the Worldwide Protein Data Bank (wwPDB, and see Table S2 for the pdb code).

### Antiproliferative assays in cancer cells

Human T-cell leukemia (Jurkat), human B-cell leukemia (RS 4,11) and human promyelocytic leukemia (HL-60) cells were grown in RPMI-1640 medium (Gibco, Milan, Italy). Breast adenocarcinoma (MCF-7 and MDA-MB-231), human non-small cell lung carcinoma (A549), human cervix carcinoma (HeLa), human prostate adenocarcinoma (PC-3), and human colon adenocarcinoma (HT-29) cells were grown in DMEM medium (Gibco, Milan, Italy). Both media were supplemented with 115 units/mL of penicillin G (Gibco, Milan, Italy), 115 μg/mL of streptomycin (Invitrogen, Milan, Italy) and 10% fetal bovine serum (Invitrogen, Milan, Italy). Stock solutions (10 mM) of the different compounds were obtained by dissolving them in DMSO. Individual wells of 96-well tissue-culture microtiter plates were inoculated with 100 μL of complete medium containing 8 × 10^3^ cells. The plates were incubated at 37 °C in a humidified 5% CO_2_ incubator for 18 h prior to the experiments. After medium removal, 100 μL of fresh medium containing the test compound at different concentrations was added to each well and incubated at 37 °C for 72 h. The percentage of DMSO in the medium in no case exceeded 0.25%. Cell viability was assayed by the (3-(4,5-dimethylthiazol-2-yl)-2,5-diphenyl tetrazolium bromide test as previously described^56^. The GI_50_ was defined as the compound concentration required to inhibit cell proliferation by 50%, in comparison with cells treated with the maximum amount of DMSO (0.25%) and considered as 100% viability. In additional experiments, cell viability was also determined by calculating the values of trypan blue positive cells (dead cells) and trypan blue negative (live cells) from a mixture of the cell suspension and 0.4% trypan blue solution.

### Antiproliferative assays in non-tumoral cells

Peripheral mononuclear cells (PBMC) from healthy donors were obtained by separation on Lymphoprep (Fresenius KABI Norge AS) gradient. After extensive washing, cells were resuspended (1 × 10^6^ cells/mL) in RPMI-1640 with 10% fetal bovine serum and incubated overnight. For cytotoxicity evaluations in proliferating PBL cultures, non-adherent cells were resuspended at 5 × 10^5^ cells/mL in growth medium, containing 2.5 μg/mL PHA (Irvine Scientific). Different concentrations of the test compounds were added, and viability was determined 72 h later by the MTT test. For cytotoxicity evaluations in resting PBL cultures, non-adherent cells were resuspended (5 × 10^5^ cells/mL) and treated for 72 h with the test compounds, as described above.

Human Umbilical Vein Endothelial cells (HUVEC), were prepared from human umbilical-cord veins, as previously described^57^. The adherent cells were maintained in M200 medium added by LSGS (low serum growth supplement), containing FBS, hydrocortisone, hEGF, bFGF, heparin, gentamycin/amphotericin (Life Technologies, Monza, Italy). Once confluent, the cells were detached by trypsin–EDTA solution and used in experiments from the first to sixth passages.

Human fibroblasts from foreskin were isolated as previously described^58^ and maintained in DMEM medium with 10% fetal bovine serum added.

### Cell-cycle distribution analysis

For flow-cytometric analysis of the DNA content, 5 × 10^5^ HeLa or Jurkat cells in exponential growth were treated with different concentrations of the test compounds for 24 and 48 h. After the incubation period, the cells were collected, centrifuged, and fixed with ice-cold ethanol (70%). The cells were then treated with lysis buffer containing RNAse A and 0.1% Triton X-100, and then stained with propidium iodide (PI). Samples were analyzed in a Cytomics FC500 flow cytometer (Beckman Coulter). DNA histograms were analyzed using MultiCycle for Windows (Phoenix Flow Systems).

### Annexin-V/PI assay

Surface exposure of PS on apoptotic cells was measured by flow cytometry with Cytomics FC500 (Beckman Coulter) by adding simultaneously annexin-V conjugated to fluorescein isothiocyanate (FITC) and PI to cells according to the manufacturer′s instructions (Annexin-V Fluos, Roche Diagnostic).

## Additional Information

**How to cite this article**: Schiaffino-Ortega, S. *et al.* Design, synthesis, crystallization and biological evaluation of new symmetrical biscationic compounds as selective inhibitors of human Choline Kinase α1 (ChoKα1). *Sci. Rep.* **6**, 23793; doi: 10.1038/srep23793 (2016).

## Supplementary Material

Supplementary Information

## Figures and Tables

**Figure 1:**
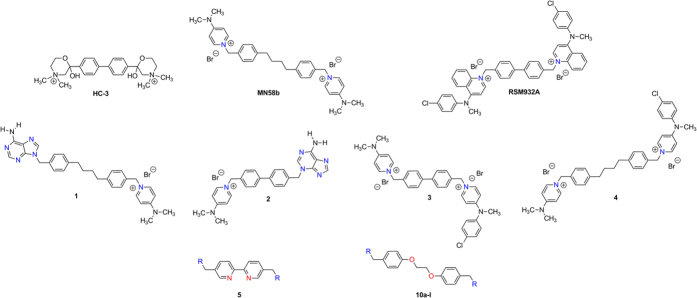
Structure of compounds **HC-3**, **MN58b**, **RSM932A** and structures of symmetrical and non-symmetrical inhibitors of choline kinase previously published (compounds **1–5**). General structure of compounds **10a-l** described in this paper.

**Table 1:** Kd values of selected compounds evaluated by tryptophan fluorescence quenching.

Compound	HsChoKα1 Kd (μM)^a^
**10a**	0.700 ± 0.080
**10f**	0.295 ± 0.127
**10g**	0.517 ± 0.099
**10k**	0.241 ± 0.03
**10l**	0.357 ± 0.039
**4**	0.110 ± 0.01
**HC-3**	0.18 ± 0.03

^a^Kd values of indicated compounds for ChoKα1 are expressed as mean ± S.D. of at least three independent experiments.

**Table 2:** *In vitro* inhibitory effects of compounds 10a-l.

Comp.	clogP Ann 2005	^a^IC50 (μM) ChoKα1 purified	Antiproliferative activity ^b^GI50 (μM)								
			HeLa	HT−29	Jurkat	HL-60	RS4,11	MCF-7	PC-3	A549	MDA-MB-231
10a	−0.36	1.00 ± 0.01	0.079 ± 0.024	0.11 ± 0.01	0.12 ± 0.08	0.10 ± 0.04	0.045 ± 0.005	0.092 ± 0.019	0.051 ± 0.01	0.027 ± 0.010	0.10 ± 0.05
10b	0.36	9.56 ± 1.45	0.082 ± 0.041	4.3 ± 0.42	0.068 ± 0.016	0.042 ± 0.005	1.12 ± 0.22	0.17 ± 0.032	4.5 ± 1.1	2.3 ± 1.1	0.09 ± 0.02
**10c**	1.8	1.63 ± 0.14	5.6 ± 0.25	4.3 ± 1.1	7.3 ± 0.50	2.1 ± 0.6	2.7 ± 0.13	3.5 ± 0.72	3.1 ± 0.7	4.9 ± 0.7	2.2 ± 0.27
**10d**	−1.01	37.54 ± 4.45	16.9 ± 2.5	>100	>100	84.1 ± 7.4	37.5 ± 4.2	91.4 ± 7.6	94.3 ± 12.4	61.3 ± 16.7	74.3 ± 2.6
**10e**	−2.42	9.51 ± 1.14	>100	94.0 ± 4.2	>100	>100	66.0 ± 6.6	50.9 ± 11.7	48.0 ± 1.1	>100	63.2 ± 3.9
**10f**	3.02	6.85 ± 0.81	0.15 ± 0.031	0.12 ± 0.017	0.060 ± 0.002	0.063 ± 0.013	0.24 ± 0.05	0.17 ± 0.065	0.47 ± 0.12	0.21 ± 0.06	0.001 ± 0.001
**10g**	3.56	3.27 ± 0.66	0.25 ± 0.06	0.75 ± 0.12	0.098 ± 0.031	0.71 ± 0.24	0.15 ± 0.04	0.46 ± 0.063	0.26 ± 0.01	0.11 ± 0.018	0.19 ± 0.009
**10h**	3.82	2.79 ± 0.17	0.32 ± 0.062	0.35 ± 0.07	0.31 ± 0.07	0.92 ± 0.06	0.026 ± 0.006	0.32 ± 0.04	0.85 ± 0.32	0.18 ± 0.08	0.23 ± 0.04
**10i**	4.23	16.22 ± 0.44	1.5 ± 0.73	1.0 ± 0.15	0.76 ± 0.26	0.87 ± 0.28	0.50 ± 0.09	0.66 ± 0.07	0.72 ± 0.09	0.29 ± 0.10	0.15 ± 0.05
**10j**	2.27	1.66 ± 0.09	0.17 ± 0.084	0.15 ± 0.08	0.11 ± 0.026	0.42 ± 0.22	0.16 ± 0.012	0.11 ± 0.047	0.09 ± 0.04	0.30 ± 0.068	0.01 ± 0.005
**10k**	2.76	2.02 ± 0.08	0.26 ± 0.074	0.27 ± 0.05	0.072 ± 0.013	0.18 ± 0.06	0.036 ± 0.008	0.28 ± 0.09	0.11 ± 0.028	0.52 ± 0.02	0.061 ± 0.003
**10l**	2.36	0.92 ± 0.01	0.37 ± 0.18	0.56 ± 0.21	0.007 ± 0.003	0.16 ± 0.07	0.42 ± 0.14	0.022 ± 0.007	0.8 ± 0.2	0.14 ± 0.06	0.05 ± 0.02
**MN58b**	0.01	0.78 ± 0,03	1.9 ± 0.1	1.9 ± 0.4	0.35 ± 0.1	0.32 ± 0.03	1.0 ± 0.3	1.8 ± 0.06	n.d.	0.54 ± 0.2	0.31 ± 0.12
**RSM932A**	2.92	1.92 ± 0,06	0.83 ± 0.1	0.4 ± 0.2	0.41 ± 0.1	0.93 ± 0.1	0.17 ± 0.04	0.18 ± 0.10	n.d.	0.45 ± 0.09	0.17 ± 0.05

^a^IC_50_ = Compound concentration required to inhibit ChoKα1 enzyme by 50%.

^b^GI_50_ = Compound concentration required to inhibit tumor-cell proliferation by 50%.

Values are the mean ± SEM for three independent experiments. n.d. not determined.

**Table 3:** *In vitro* inhibitory effects of selected compounds in non-tumoral cells.

Antiproliferative activity^a^ GI_50_ (μM)				
Comp	PBL(resting)	PBL(+Pha)^b^	Human fibroblasts	HUVEC
**10a**	1.5 ± 0.64	0.034 ± 0.007	5.8 ± 1.3	5.1 ± 0.43
**10b**	34.8 ± 15.6	1.88 ± 0.71	30.5 ± 9.6	n.d.
**10f**	1.4 ± 0.6	0.10 ± 0.03	9.8 ± 2.5	n.d.
**10g**	0.98 ± 0.25	0.55 ± 0.11	7.4 ± 2.4	10.4 ± 3.5
**10h**	1.0 ± 0.42	0.32 ± 0.03	3.7 ± 0.43	16.0 ± 6.6
**10j**	2.0 ± 0.24	0.095 ± 0.021	14.3 ± 1.2	n.d.
**10k**	2.1 ± 0.80	0.034 ± 0.007	3.2 ± 0.86	n.d.
**10l**	3.8 ± 0.55	0.49 ± 0.15	9.6 ± 1.3	11.4 ± 5.9
**RSM932A**	1.1 ± 0.09	0.23 ± 0.05	n.d	0.46 ± 0.047
**MN58b**	2.0 ± 0.4	0.15 ± 0.05	n.d.	2.1 ± 0.58

^a^GI_50_ = Compound concentration required to inhibit tumor-cell proliferation by 50% Values are the mean ± SEM for three independent experiments. n.d. not determined.

^b^Pha, Phytohematoagglutini.
