# Supplementary material for: Design, synthesis, crystallization and biological evaluation of new symmetrical biscationic compounds as selective inhibitors of human Choline Kinase α1 (ChoKα1)
Source: Sci Rep. 2016 Mar 31;6:23793. doi: 10.1038/srep23793 (PMC4814829; doi:10.1038/srep23793)
Supplement: Supplementary Information [file srep23793-s1.pdf]

Supplementary information

Design, synthesis, crystallization and biological evaluation of new symmetrical biscationic compounds as selective inhibitors of human Choline Kinase α1 (ChoKα1)

Santiago Schiaffino-Ortega,[1‡] Eleonora Baglioni,[1‡], Elena Mariotto,[3] Roberta Bortolozzi,[3] Lucía Serrán-Aguilera,[1,4] Pablo Ríos-Marco,[2] Maria Paz Carrasco-Jimenez,[2], Miguel A. Gallo,[1] Ramon Hurtado-Guerrero,[4] Carmen Marco,[2] Giuseppe Basso,[3] Giampietro Viola,[3]*Antonio Entrena[1]*, Luisa Carlota López-Cara,[1]*

[1]Departamento de Química Farmacéutica y Orgánica, Facultad de Farmacia, Campus de Cartuja, 18071 Granada (Spain). [2]Departamento de Bioquímica y Biología Molecular I, Facultad de Ciencias, Campus Fuentenueva, 18071 Granada (Spain). [3]Dipartimento di Salute della Donna e del Bambino, Laboratorio di Oncoematologia, Università di Padova, Padova, Italy. [4]Institute of Biocomputation and Physics of Complex Systems (BIFI), University of Zaragoza, BIFI-IQFR (CSIC) Joint Unit, Mariano Esquillor s/n, Campus Rio Ebro, Edificio I+D; Fundacion ARAID, Edificio Pignatelli 36 (Spain)

To whom correspondence should be addressed: *Email: lcarlotalopez@ugr.es. Phone: (+)34-958-243849. Fax: (+)34-958-243850. *Email: aentrena@ugr.es. Phone: (+)34-958-243848, Fax: (+)34-958-243850. * Email: giampietro.viola.1@unipd.it. Phone: (+)39 (0) 49 8211451. Fax: +39 (0) 49 8211462

CONTENTS

a) **Table S1.** Experimental conditions under microwaves.
b) **Figure S1.**Detail view of compounds **2** and **4** inserted into of Cho binding site.
c) **Figure S2.**Resulting poses of compounds **10b**, **10d**, and **10e** in the ChoKα1/**2**. .
d) **Figure S3.**Resulting poses of compounds **10c**, **10f-k-d** in the of ChoKα1/**4**
e) **Figure S4.** Quenching of intrinsic ChoKα1 tryptophan fluorescence.
f) **Figure S5.** Jurkat cells treated with the indicated concentration of **10a** for 72h and then incubated for further 72 h in the absence of compound.
g) **Figure S6.** Comparison of the docking pose of compound **10a** with the one of the crystal structure.
h) **Table S2.** Data collection and refinement statistics.
i) Experimental Procedures
j) References

Table S1: Conditions reaction to obtain compound **7** and **8**

Synthesis of **7**			
	Quantity	Time and Temperature	Yields
Refluxed	5 g of **6**	8 h - 70 °C	21%
MW	1 g of **6**	28'- 130 °C	20%
		14'- 140 °C	31%
		28'- 140 °C	35%
		56'- 140 °C	27%
		14'- 150 °C	25%
Synthesis of **8**			
Refluxed	1 g of **7**	5 h - 70 °C	39%
MW	100 mg of **7**	21'- 120 °C	65%
	200 mg of **7**	21'- 120 °C	52%
	300 mg of **7**	21'- 120 °C	55%
		40'- 120 °C	50%

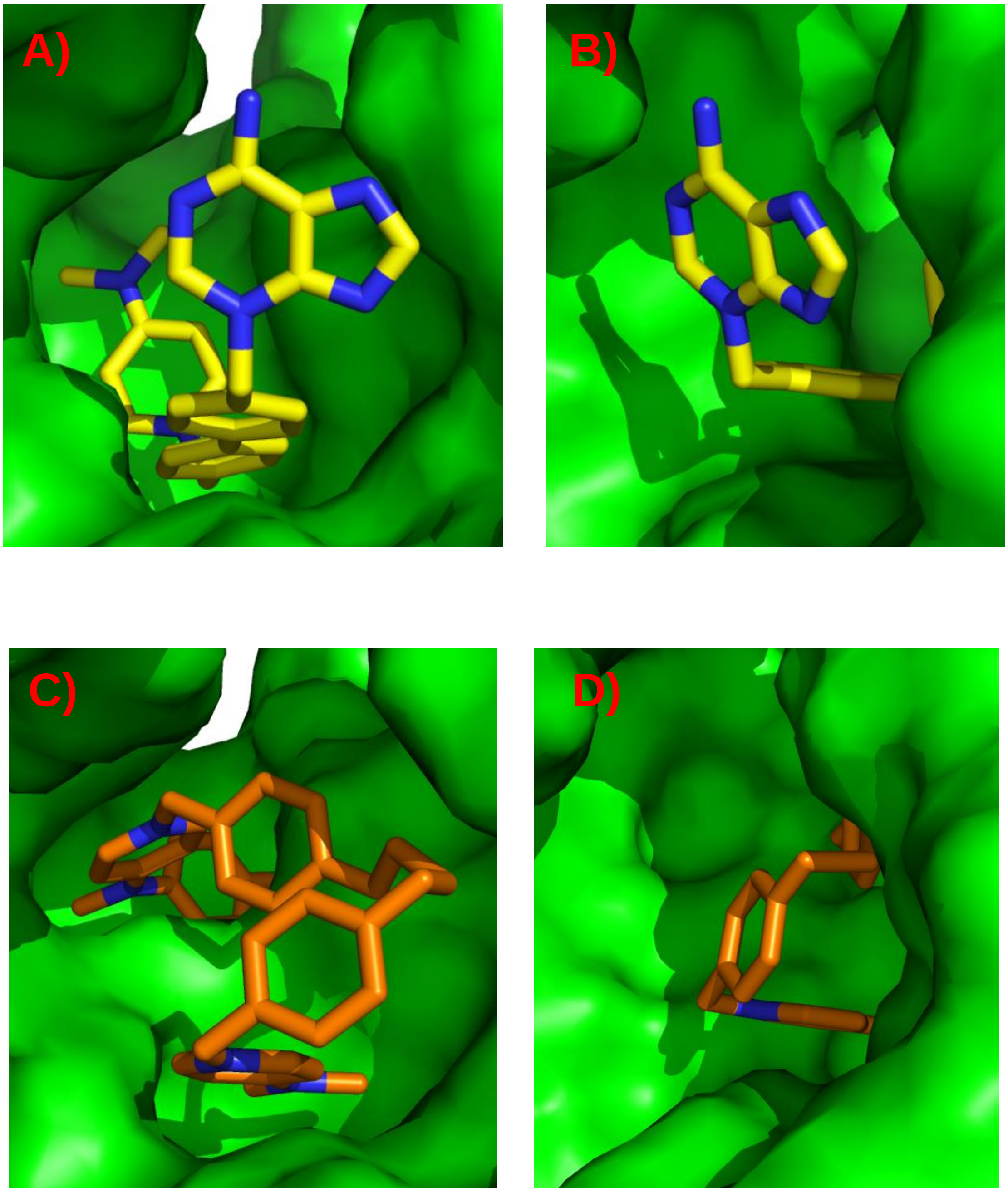

Figure S1. Detail view of the compounds **2** (carbon atoms in yellow color) and **4** (carbon atoms in cyan color) inserted into the Cho binding site represented by the protein surface. (**A**) The 1-(biphenyl-4-ylmethyl)-4-(dimethylamino)pyridinium fragment of compound **2** is inserted into the Cho binding site. (**B**) The adenine moiety is situated outside of the Cho binding site, and this fact justified the absence of interactions between the adenine moiety and the protein. (**C**) The 4-chloro-*N*-methylaniline fragment is inserted into the additional binding site, and the rest of the molecule is inserted into the ChoK binding site. (**D**) The second cationic head is also situated inside the protein.

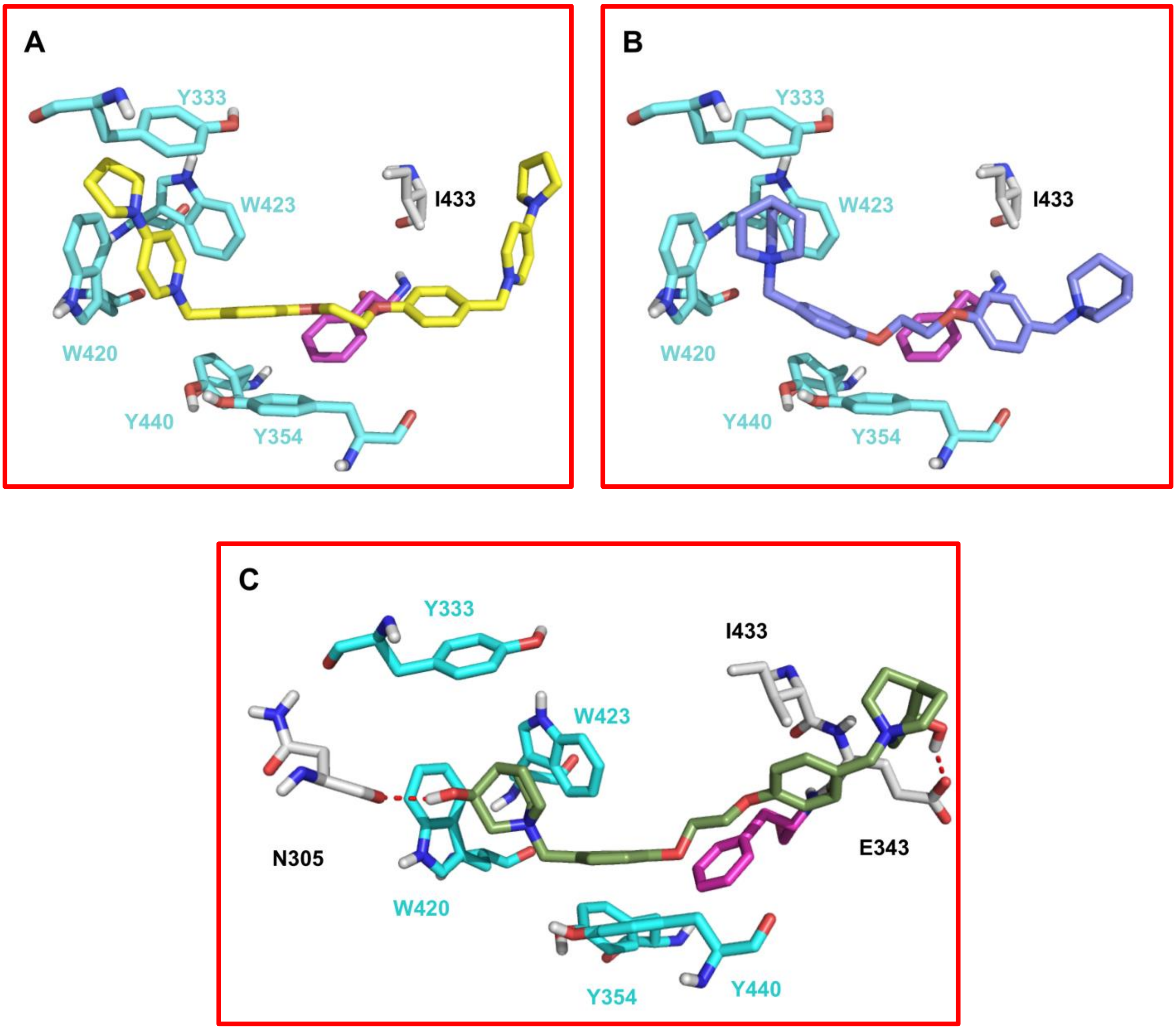

Figure S2. Poses resulting in the docking studies of compounds **10b** (**A**, carbon atoms in yellow color), **10d** (**B**, carbon atoms in purple color) and **10e** (**C**, carbon atoms in green color) inside the Cho binding site of ChoKα1/**2** complex (PDB ID: 4BR3)

.

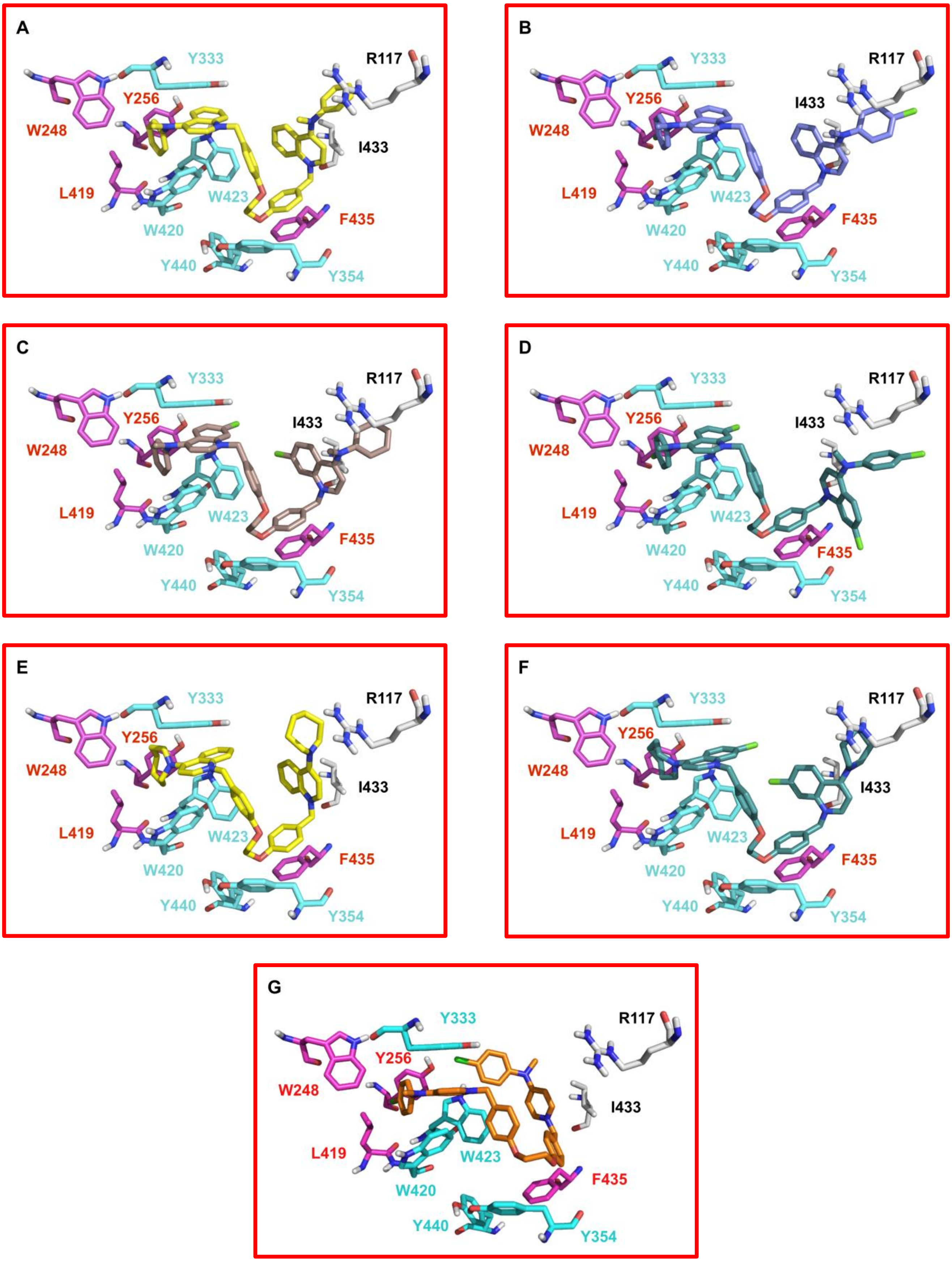

Figure S3. Poses resulting in the docking studies of compounds **10f** (**A**, carbon atoms in yellow color), **10g** (**B**, carbon atoms in blue-slate color), **10h** (**C**, carbon atoms in reddish-salmon color), **10i** (**D**, carbon atoms in blue color), **10j** (**E**, carbon atoms in yellow color), **10k (F,** carbon atoms in blue color**)** and **10c** (carbon atoms in orange color) inside the Cho binding site of ChoKα1/**4** complex (PDB ID: 4CG8).

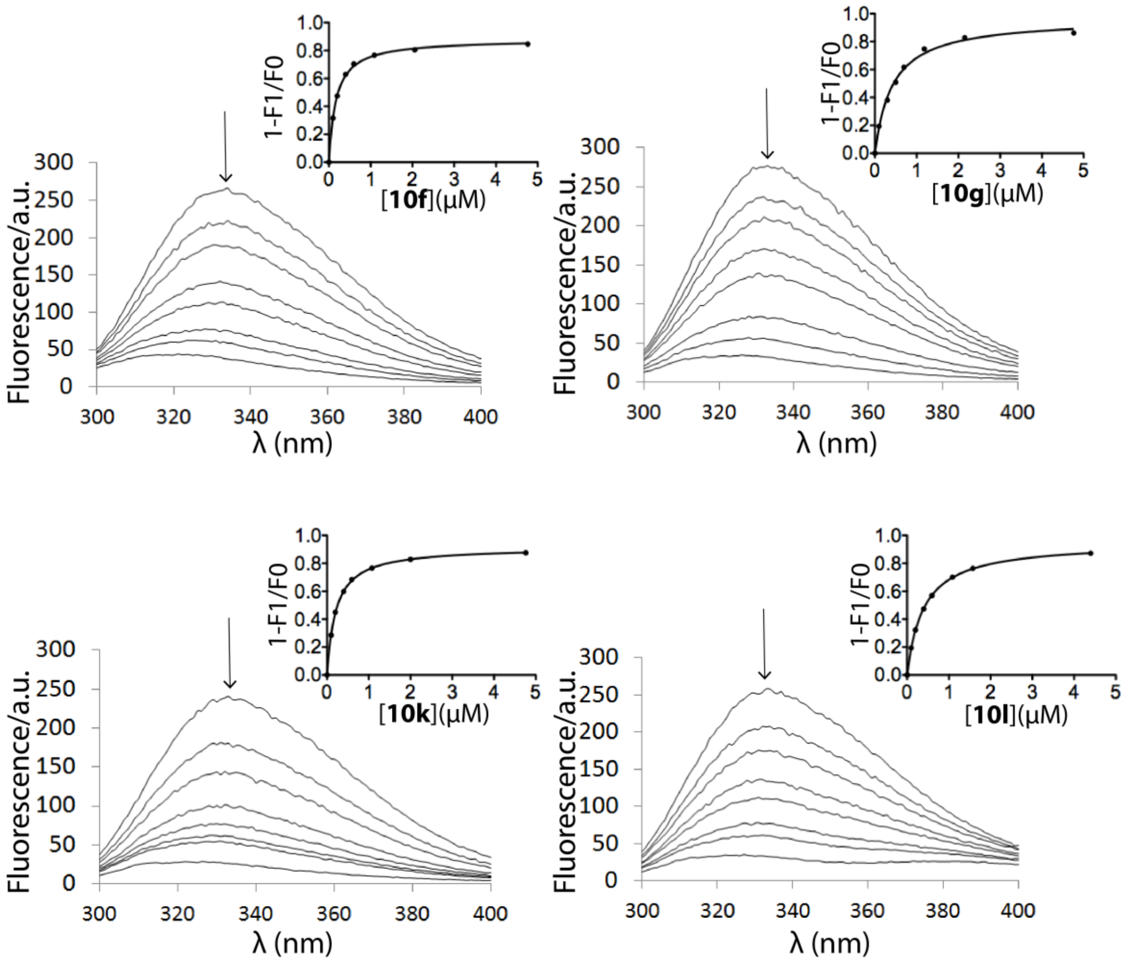

Figure S4: Quenching of intrinsic ChoKα1 tryptophan fluorescence measured at increasing concentrations of compounds **10f, 10g, 10k** and **10l.** All data points represent the means ± S.D. for three measurements. The K_d for the four compounds was determined by fitting fluorescence intensity data against its concentration. A black arrow indicates the decrease of the maximal fluorescence signal with increasing concentrations of each compound.

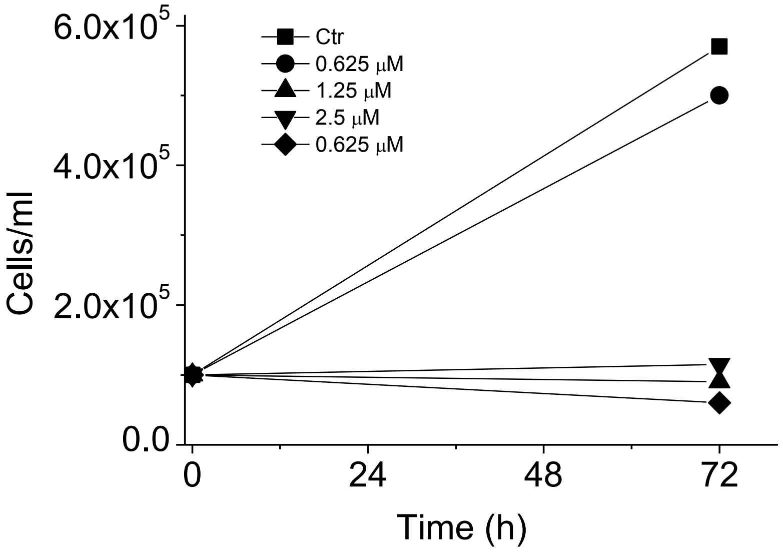

Figure S5

Jurkat cells were treated with the indicated concentration of **10a** for 72 h and then cells were harvested, washed, counted, and incubated in a drug-free medium. After a further 72 h of incubation, cell viability was analyzed by trypan blue exclusion assay as described in the experimental section.

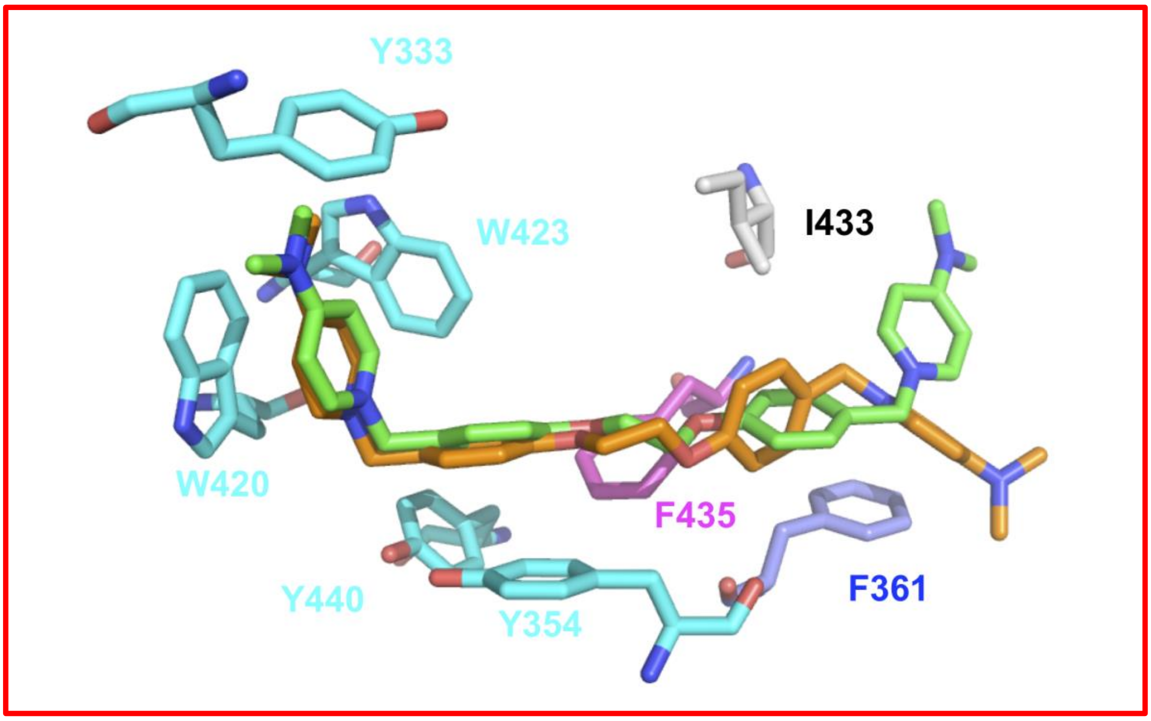

Figure S6. Superposition of the resulting pose of compound **10a** (carbon atoms in green color) with compound **10a** (carbon atoms in orange color) inserted into Cho binding site into the crystal structure (PDB ID: 4TST)

Table S2. Data collection and refinement statistics**.** Values in parentheses refer to the highest resolution shell. Ramachandran plot statistics were determined with PROCHECK.

	**ChoKα1-10a**
Space group	$P4_32_12$
Wavelength (Å)	1.0507
Resolution (Å)	58-1.45 (1.53-1.45)
Cell dimensions (Å)	*a* =60.65<br>*b* = 60.65<br>*c* = 220.40
Unique reflections	74.167
Completeness	99.9 (99.4)
R_{sym}	0.058 (0.672)
I/s(*I*)	34.8 (5.0)
Redundancy	23.2 (14.0)
R_{work} / R_{free}	0.197/0.218
RMSD from ideal geometry, bonds (Å)	0.0048
RMSD from ideal geometry, angles (º)	1.079
<*B*> protein (Å^2)	18.11
<*B*> EB3D (Å^2)	30.50
<*B*> solvent (Å^2)	33.42
<*B*> Ethylenglycol (Å^2)	29.97
Ramachandran plot:<br>Most favored (%)<br>Additionally allowed (%)<br>Generously allowed (%)	 97.61<br>1.882.09<br>0.000.30
PDB ID	4TST

Experimental Procedures

General Experimental details

Chemistry. Melting points were taken in open capillaries on a Stuart Scientific SMP3 electrothermal melting-point apparatus and were uncorrected. Elemental analyses were performed on the Thermo Scientific Flash 2000 analyzer only on the final compounds tested as *Hs*ChoKα1 inhibitors. The measured values for C, H, and N agreed to within ± 0.40% of the theoretical values. Analytical thin-layer chromatography (TLC) was performed using Merck Kieselgel 60 F254 aluminum plates and visualized by UV light or iodine. All evaporation occurred *in vacuo* with a Büchi rotary evaporator and the pressure controlled by a Vacuubrand CVCII apparatus. For flash chromatography, Merck silicagel 60 with a particle size of 0.040 – 0.063 mm (230 – 400 mesh ASTM) was used: 600 MHz ^{1}H and 151 MHz ^{13}C NMR Varian Direct Drive; a 400 MHz ^{1}H and 100 MHz ^{13}C NMR Varian Direct Drive; or 300 MHz ^{1}H and 75 MHz ^{13}C NMR Varian Inova Unity spectrometers at room temperature. Chemical shifts (δ) are quoted in parts per million (ppm) and are referenced to the residual solvent peak. Spin multiplicities are given as s (singlet), bs (broad singlet), d (doublet), t (triplet), and m (multiplet). High-resolution Nano-Assisted Laser Desorption/Ionization (NALDI-TOF) or Electrospray Ionization (ESI-TOF) mass spectra were carried out on a Bruker Autoflex or a Waters LCT Premier Mass.

General procedure A for the synthesis of the linker

1,2-bis(*p*-tolyloxy)ethane (**7**) [1]: To a solution of 0.4 g of NaOH in 5 mL of EtOH 1 g of 4-methoxyphenol (**6**) was added. The mixture was stirred for 30 min at room temperature and afterwards 0.4 mL of 1,2-dibromoethane was added before microwave irradiation at 140 ºC for 28 min. The reaction was quenched by cooling (ice/water bath) and by addition of 25 mL distilled water. The solid was filtered

and washed twice with water H_2O (2 x 2 mL) and EtOH (1 x 2 mL), dried to vacuum to furnished a white solid identified as 1,2-bis(*p*-methylphenoxy)ethane (**7**). Yield 35%.

^{1}H NMR (300 MHz, $CDCl_3$) δ: 7.10-7.08 (d, *J* = 8.61 Hz, 4H), 6.86-6.84 (d, *J* = 8.44 Hz, 4H), 4.28 (s, 4H), 2.29 (s, 6H).

1,2-bis(4-(bromomethyl)phenoxy)ethane (**8**) [2]: To a solution of **7** (0.1, 25 mmol) in tetrachloromethane (3 mL), a mixture of dibenzoylperoxide (0.5 mg, 2 mmol) and NBS (9.08 mg, 51 mmol) was added. The mixture was purged with argon before microwave irradiation at 120 ºC, 21 min. The precipitate was filtered and washed twice with diethylether (2 x 2 mL), and dried under vacuum to give **7** as a yellow solid. Yield: 65%,

^{1}H NMR (300 MHz, $CDCl_3$) δ: 7.35-7.32 (d, *J* = 8.73 Hz, 4H), 6.92-6.88 (d, *J* = 8.73 Hz, 4H), 4.50 (s, 4H), 4,32 (s, 4H).

General procedure B for the synthesis of the cationic heads

The cationic heads (**9a-d**) are commercially available while that the cationic heads (**9f-l**) were obtained following the procedure previously described [3-9], starting of 4-chloroquinoline and 7,4-dichloroquinoline which were treated with *N*-methylanilene, 4-chloro-*N*-methylaniline, perhydroazepine or pyrrolidine to obtain **9e** (4-*N*-methylanilinoquinoline), **9f** (4-(4-chloro-*N*-methylanilino)quinoline), **9g** (7-chloro-4-(*N*-methylanilino)quinoline), **9h** (7-chloro-4-(4-chloro-*N*-methylanilino)quinoline), **9i** (4-perhydroazepinoquinoline), **9j** (7-chloro-4-perhydroazepinoquinoline) and/or **9k** (7-chloro-4 (pyrrolidino)quinoline).

References

1. Cantrill, S.J. *et al.* The influence of macrocyclic polytether constitution upon ammmonium ion/crown ether recognition proceses *Chem. Eur. J.* **6**, 2274-2287 (2000).

2. Xiao, J. *et al.* Binuclear titanocenes linked by the bridge combination of rigid and flexible segment: Synthesis and their use as catalysts for ethylene polymerization. *Journal of Molecular Catalysis A: Chemical*. **267**, 86-91 (2007).

3. Gómez-Pérez, V. *et al.* Bis-pyridinium and Bis-quinolinium Derivatives as Choline Kinase Inhibitors with Antiproliferative Activity against the Human Breast Cancer SKBR-3 Cell Line. *Chem. Med. Chem.* **7**, 663 – 669 (2012).

4. Campos, J. *et al.* QSAR of 1,1'-(1,2-Ethylenebisbenzyl)bis(4-substitutedpyridinium) dibromides as choline kinase Inhibitors: A different approach for antiproliferative drug design. *Bioorg. & Med. Chem. Lett.* **10**, 767-770 (2000).

5. Campos, J. M. *et al.* Quantitative Structure Activity Relationships for a Series of Symmetrical Bisquaternary Anticancer compounds. *Bioorg. & Med. Chem.* **10**, 2215-2231 (2002).

6. Sánchez-Martín, R.M. *et al.* Symmetrical bis-quinolinium compounds: New human choline kinase inhibitors with antiproliferative activity against the HT-29 cell line. *J. Med. Chem.* **48**, 3354-3363. (2005).

7. Conejo-García, A. *et al.* Choline kinase inhibitory effect and antiproliferative activity of new 1,1',1''-(benzene-1,3,5-triylmethylene)tris{4-[disubstituted)amino]pyridinium} tribromides. *Eur. J. Med. Chem*. **38**, 109-116 (2002).

8. Conejo-García, A. *et al.* Bispyridinium cyclophanes: novel templates for human choline kinase inhibitors. *J. Med. Chem*. **46**, 3754-3757 (2003).

9. Castro-Navas, F.F. *et al.* New more polar symmetrical bipyridinic compounds: new strategy for the inhibition of choline kinase α1. *Future in Medicinal Chemistry*. **7**, 417-436. (2015)
